# ChEMU 2020: Natural Language Processing Methods Are Effective for Information Extraction From Chemical Patents

**DOI:** 10.3389/frma.2021.654438

**Published:** 2021-03-25

**Authors:** Jiayuan He, Dat Quoc Nguyen, Saber A. Akhondi, Christian Druckenbrodt, Camilo Thorne, Ralph Hoessel, Zubair Afzal, Zenan Zhai, Biaoyan Fang, Hiyori Yoshikawa, Ameer Albahem, Lawrence Cavedon, Trevor Cohn, Timothy Baldwin, Karin Verspoor

**Affiliations:** ^1^The University of Melbourne, Parkville, VIC, Australia; ^2^RMIT University, Melbourne, VIC, Australia; ^3^VinAI Research, Hanoi, Vietnam; ^4^Elsevier BV, Amsterdam, Netherlands; ^5^Elsevier Information Systems GmbH, Frankfurt, Germany; ^6^Fujitsu Laboratories Ltd., Tokyo, Japan

**Keywords:** named entity recognition, event extraction, information extraction, chemical reactions, patent text mining, cheminformatics

## Abstract

Chemical patents represent a valuable source of information about new chemical compounds, which is critical to the drug discovery process. Automated information extraction over chemical patents is, however, a challenging task due to the large volume of existing patents and the complex linguistic properties of chemical patents. The Cheminformatics Elsevier Melbourne University (ChEMU) evaluation lab 2020, part of the Conference and Labs of the Evaluation Forum 2020 (CLEF2020), was introduced to support the development of advanced text mining techniques for chemical patents. The ChEMU 2020 lab proposed two fundamental information extraction tasks focusing on chemical reaction processes described in chemical patents: (1) *chemical named entity recognition*, requiring identification of essential chemical entities and their roles in chemical reactions, as well as reaction conditions; and (2) *event extraction*, which aims at identification of event steps relating the entities involved in chemical reactions. The ChEMU 2020 lab received 37 team registrations and 46 runs. Overall, the performance of submissions for these tasks exceeded our expectations, with the top systems outperforming strong baselines. We further show the methods to be robust to variations in sampling of the test data. We provide a detailed overview of the ChEMU 2020 corpus and its annotation, showing that inter-annotator agreement is very strong. We also present the methods adopted by participants, provide a detailed analysis of their performance, and carefully consider the potential impact of data leakage on interpretation of the results. The ChEMU 2020 Lab has shown the viability of automated methods to support information extraction of key information in chemical patents.

## 1. Introduction

Discovery and development of new drugs is continually needed by our society. New drugs are required by our healthcare systems to address unmet medical needs, and pharmaceutical industries strive to bring better drugs to market. However, the development of new drugs is an expensive process, which may take more than 10 years and cost more than 2.6 billion dollars, with a success rate of <12%^1^. In order to accelerate the process, reduce the overall cost, and improve the success rate of novel formulations, there has been an increasing interest in leveraging artificial intelligence techniques for drug discovery (Smalley, 2017; Mak and Pichika, 2019).

Artificial intelligence techniques may benefit the drug development process in various ways. One active research area is the development of information extraction tools over chemical literature. Chemical literature contains valuable information about the latest advancements in the chemistry domain that is important to make findable and accessible. However, due to the rapid growth in chemical literature, new discoveries are easily missed while manual extraction of this information is increasingly infeasible (Muresan et al., 2011). Therefore, the development of automatic information extraction systems over chemical literature has attracted extensive research interest. The main idea in this work is to leverage natural language processing (NLP) techniques to build systems that can automatically and effectively process lengthy chemical texts, including scientific literature or patents, in order to extract key information out of them. The extracted information can be used directly for related research, such as drug target ranking (Hamed et al., 2021), or to construct a structured knowledge base that can be searched.

Chemical patents are acknowledged as a critical source of information about new discoveries in chemistry. Discoveries of new chemical compounds are typically disclosed in chemical patents (Bregonje, 2005; Senger et al., 2015) and patents may lead other chemical literature, such as scientific journals by up to 3 years. Moreover, some information about new chemical compounds, e.g., their detailed synthesis processes, is exclusively provided in chemical patents. These details are important for understanding the compound prior art, and provide a means for novelty checking and validation (Akhondi et al., 2014, 2019).

Due to the significant value of information in chemical patents, many research efforts have been made toward the development of more effective information extraction systems specifically for chemical patents (Parapatics and Dittenbach, 2011; Akhondi et al., 2014; Chen et al., 2020). Several fundamental information extraction tasks, such as named entity recognition (NER) (Zhai et al., 2019), and relation extraction (Peng et al., 2018) have been extensively investigated. There also exist several shared tasks that focus on information extraction in the chemistry domain, such as ChemDNER (Krallinger et al., 2015a,b).

The ChEMU (Cheminformatics Elsevier Melbourne University) lab is an initiative to encourage research on methods for automated information extraction from chemical patents. As a first running of ChEMU, ChEMU2020 lab focused on extraction of *chemical reactions* from patents (He et al., 2020a; Nguyen et al., 2020). We prepared two fundamental information extraction tasks. Task 1—named entity recognition (NER)—focused on identifying the set of *named entities* that are essential to describe chemical reaction process. Task 2—event extraction (EE)—addressed identifying the sequence of *event steps* in a chemical reaction which transforms the starting material to the reaction compound. Compared with existing shared tasks, such as BioNLP, we primarily focus on information extraction in the context of chemical patents rather than scientific literature, which introduces some challenges due to the complex linguistic properties of patents (Zhai et al., 2019). Compared with the shared task ChemDNER (Krallinger et al., 2015a), which also addressed chemical named entity recognition, we go beyond the scope of entity mentions and chemical entity passage detection, and require the identification of the specific roles of chemical entities within reactions, such as whether the chemicals serve as starting materials or products. Moreover, we consider data from full patent texts in our task, instead of solely focusing on titles and abstracts of patents.

A high-quality new corpus was made publicly available to support the two tasks in ChEMU2020 lab. The corpus was prepared using 1,500 text segments sampled from 180 English patents from the European Patent Office and the United States Patent and Trademark Office. Three chemical experts were hired to manually annotate the corpus, labeling named entities and event steps in these text segments. Two of them reviewed all text segments independently and the third annotator acted as an adjudicator who resolved their disagreements and merged their annotations into the final gold-standard corpus. The inter-annotator-agreement (IAA) score reaches 0.9760 and 0.9506 for the two tasks, respectively.

The ChEMU2020 lab was held during April to June 2020. Three tracks were made available to participants: one track each for the NER and EE tasks individually, and a third track for end-to-end systems which address both tasks simultaneously. We received registrations of 37 teams from 13 countries in total. We received 26 runs (including one post-evaluation run) from 11 teams in Task 1, 10 runs from five teams in Task 2, and 10 runs from four teams in the third track. In this paper, we provide a detailed overview of the activities within ChEMU2020 lab, including the new ChEMU corpus, the tasks, the evaluation framework, the evaluation results, and a summary of participants' approaches. This paper is an extension of our previous overview papers (He et al., 2020a,b) and thereby the task descriptions (section 4) and core evaluation results (section 5) are repeated here from those papers. Our focus is to provide additional detail about the preparation of the corpus we developed (section 3) and to provide more comprehensive analysis of the evaluation results. The corpus is available for use (Verspoor et al., 2020); the test data can be submitted for evaluation through the shared task website at http://chemu2020.eng.unimelb.edu.au/.

## 2. Related Work

To assess and advance the natural language processing (NLP) techniques in the biochemical domain, many shared tasks/labs have been organized, including n2c2^2^, TREC^3^, BioCreative^4^, BioNLP^5^, and CLEF workshops^6^. These shared tasks have covered a range of benchmark text mining tasks: *information retrieval*, such as document retrieval [CLEF eHealth 2014 (Kelly et al., 2014)] and text classification [CoNLL 2010 (Farkas et al., 2010)]; *word semantics*, such as named entity recognition [BioCreative II (Morgan et al., 2008) Task 1] and mention normalization [BioCreative III (Arighi et al., 2011; Lu et al., 2011) Gene Normalization Task]; *relation semantics*, such as event extraction [GENIA Event Extraction (Kim et al., 2013)] and interaction extraction [Drug-Drug Interaction (Herrero-Zazo et al., 2013)]; and *high-level applications*, such as question answering [Semantic QA (Tsatsaronis et al., 2012)] and document summarization [Biomed-Summ (Jaidka et al., 2016)].

Nevertheless, most of these shared tasks/labs did not focus on the domain of chemical patents. These shared tasks mainly focused on the text mining over biomedical texts (e.g., scientific literature, such as PubMed abstracts) or clinical data (e.g., clinical health records). Text mining techniques that are developed for biomedical or biochemical texts, such as scientific journals and clinical records may not be effective for chemical patents. This is because their purpose is distinct—chemical patents are written for protection of intellectual property related to chemical compounds—and their content has different scope and characteristics, including variations in linguistic structures. Thus, it is critical to develop text mining techniques that are tailored for chemical patents.

Only two shared tasks have previously considered chemical patents. TREC 2009 (Lupu et al., 2009) provided a chemical information retrieval track for the tasks of *ad hoc* retrieval of chemical patents and prior art search. However, this track differs significantly from the subtasks in our ChEMU lab: it addresses document-level retrieval and relevance to queries instead of considering the detailed content of each document. The ChemDNER-patents task (Krallinger et al., 2015c) at the BioCreative V workshop was the task that is most similar with ours. It aimed at detection of chemical compounds and genes/proteins in patent text. However, the ChemDNER-patents task only considered entity detection within patent abstracts while we consider data extracted from the full texts of patents. Moreover, our definition of chemical compound entities is much richer as our label set defines not only that a chemical or drug compound is mentioned, but also what its specific role is with respect to the chemical reaction that it is related to in the description, e.g., starting material, catalyst, or product.

ChEMU lab 2020 also contributes a new corpus on chemical text mining for the research community^7^. Most existing benchmark datasets for biochemical text mining focus on biomedical texts, i.e., texts that consider the interaction of chemicals with molecular biology or human disease. CHEMProt (Krallinger et al., 2017a) consists of 1,820 PubMed^8^ abstracts with chemical-protein interactions, DDI extraction 2013 corpus (Herrero-Zazo et al., 2013) is a collection of 792 texts selected from the DrugBank database^9^ and other 233 PubMed abstracts, and BC5CDR is a collection of 1,500 PubMed titles and abstracts selected from the CTD-Pfizer corpus, just to give a few examples.

The number of public datasets that focus on the chemistry domain is limited. Further, several existing chemical datasets are based on structured/semi-structured texts rather than free, natural language, texts. For example, the ZINC 15 250k corpus^10^ is a collection of 250,000 molecules with their Simplified Molecular Input Line Entry System (SMILES) strings. The Tox21 dataset contains roughly 7,000 molecules and typical 120 characteristics, such as atomic number, aromicity, donor status. There are two datasets that are constructed from free patent texts: (1) the dataset released by the ChemDNER patents task and (2) the dataset created by Akhondi et al. (2014). However, these two datasets only contain entity annotations. Our chemical reaction corpus is further enriched by the relations between the annotated entities.

Despite the limited number of shared tasks on chemical patent mining, there is an increasing interest in developing information extraction models for patents in general research communities (Tseng et al., 2007; Akhondi et al., 2019; Yoshikawa et al., 2019). Various text mining techniques have been proposed for information extraction over chemical patents (Krallinger et al., 2017b), addressing fundamental NLP tasks, such as named entity recognition and relation extraction (Tseng et al., 2007; Vazquez et al., 2011; Akhondi et al., 2016; Zhai et al., 2019). Early techniques for chemical text mining, such as dictionary-based methods (Rebholz-Schuhmann et al., 2007; Hettne et al., 2009; Akhondi et al., 2016) and grammar-based methods (Narayanaswamy et al., 2002; Liu et al., 2012; Akhondi et al., 2015), heavily rely on expert knowledge in the chemical domain. Recently, machine learning-based techniques have reported state-of-the-art effectiveness in chemical text mining (Hemati and Mehler, 2019; Zhai et al., 2019). However, such techniques require a large amount of annotated text data, which still remains limited. Thus, ChEMU lab 2020 was hosted to provide an opportunity for NLP experts to develop information extraction systems over chemical patents. The new ChEMU reaction corpus was also made publicly available to all researchers as an important benchmark dataset for future research in this domain (Verspoor et al., 2020).

## 3. The ChEMU Chemical Reaction Corpus

### 3.1. Data Selection

The ChEMU chemical reaction corpus was built with the aid of Elsevier Reaxys® database. Reaxys is a rich information resource for chemical reactions, which contains detailed descriptions of chemical reactions that are extracted via an “excerption” process (Lawson et al., 2011), i.e., manual selection of information from literature sources, such as patents and scientific publications. We selected 180 English patents from the European Patent Office and the United States Patent and Trademark Office, for which information had been included in the Reaxys database. From these patents, 1,500 text segments were sampled from chemical reaction descriptions pre-identified by expert domain annotators, available as a product of the process used to populate information in Reaxys. We refer to each text segment as a patent “snippet” and use the two expressions interchangeably in the remainder of this paper. The key statistics of the sampled snippets are summarized in Table 1, including the total numbers of words and sentences in all snippets (Total), and the minimum (Min), maximum (Max), median (Median), and mean (Mean) of the number of words/sentences per snippet. Note that the numbers of words and sentences are computed using the NLTK tool^11^.

**Table 1:** Statistics of the selected snippets.

**Feature**	**Total**	**Min**	**Max**	**Median**	**Mean**
# Sentence	7,402	1	46	4	5.0
# Word	252,459	35	1,275	157	168.3

**Figure 1:**
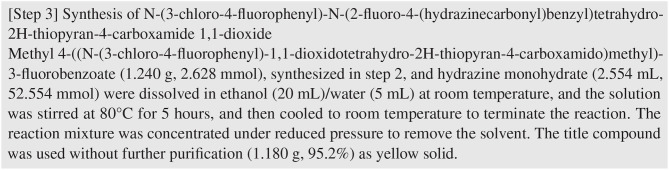
An example of one patent snippet in ChEMU chemical reaction corpus.

Figure 1 depicts an example of an extracted patent snippet in the ChEMU corpus. This example snippet describes the synthesis process of the chemical compound “N-(3-chloro-4-fluorophenyl)-N-(2-fluoro-4-(hydrazinecarbonyl)benzyl)tetrahydro-2H-thiopyran-4-carboxamide 1,1-dioxide.” The described reaction process consists of five event steps:

- Methyl 4-((N-(3-chloro-4-fluorophenyl)-1,1-dioxidotetrahydro-2H-thiopyran-4-carboxamido)methyl)-3-fluorobenzoate and hydrazine monohydrate were dissolved in ethanol at room temperature.
- The solution was stirred at 80° for 5 h.
- The solution was cooled to room temperature.
- The reaction mixture was concentrated.
- The title compound of 1.180 g, 95.2% was used without further purification.

Given the patent snippet in Figure 1, the ChEMU lab 2020 aims at identification of these five event steps, and our ChEMU chemical reaction corpus is constructed to support this target. Therefore, all patent snippets were annotated to label three types of information: (1) named entities, e.g., chemical compounds participating in these event steps; (2) trigger words signaling the occurrences of event steps; and (3) relations between trigger words and named entities that reflect how entities are involved in event steps. The first type of annotations were used to support Task 1, serving as the ground-truth entities in our evaluation. The next two types of annotations were used to support Task 2, which form the ground-truth events.

### 3.2. Annotation Guidelines

In order to obtain high-quality and consistent annotations, comprehensive annotation guidelines were prepared before the official annotation started (Verspoor et al., 2020).

#### 3.2.1. Named Entity Annotation Guidelines

As discussed, our NER task requires not only detection of named entities, but also the assignment of correct labels for detected named entities according to their roles in chemical reactions. Four categories of entities are defined: (1) chemical compounds; (2) reaction conditions; (3) yields; and (4) example labels. Furthermore, ten entity labels are defined under the four categories. In particular, five labels are defined for chemical compound entities: STARTING_MATERIAL, REAGENT_CATALYST, REACTION_PRODUCT, SOLVENT, and OTHER_COMPOUND. Two labels are defined for reaction conditions: TIME and TEMPERATURE, two labels for yields: YIELD_PERCENT and YIELD_OTHER, and another label for example labels: EXAMPLE_LABEL. The detailed definitions of the ten labels are presented in Entity Annotations in Table 2.

**Table 2:** Definitions of entity, trigger word, and relation types, i.e., labels.

**Label**	**Definition**
**Entity annotations**	
STARTING_MATERIAL	A substance that is consumed in the course of a chemical reaction providing atoms to products is considered as starting material.
REAGENT_CATALYST	A reagent is a compound added to a system to cause or help with a chemical reaction.
REACTION_PRODUCT	A product is a substance that is formed during a chemical reaction.
SOLVENT	A solvent is a chemical entity that dissolves a solute resulting in a solution.
OTHER_COMPOUND	Other chemical compounds that are not the products, starting materials, reagents, catalysts and solvents.
TIME	The reaction time of the reaction.
TEMPERATURE	The temperature at which the reaction was carried out.
YIELD_PERCENT	Yield given in percent values.
YIELD_OTHER	Yields provided in other units than %.
EXAMPLE_LABEL	A label associated with a reaction specification.
**Trigger annotations**	
REACTION_STEP	An event within which starting materials are converted into the product.
WORKUP	An event step which is a manipulation required to isolate and purify the product of a chemical reaction.
**Relation annotations**	
Arg1	The relation between an event trigger word and a chemical compound.
ArgM	The relation between an event trigger word and a temperature, time, or yield entity.

In addition to label definitions, more detailed instructions on annotations are provided in the annotation guidelines including: (1) how to correctly identify the text spans of named entities; and (2) how to assign correct labels to entities where there is ambiguity; and (3) annotation decisions for problematic examples encountered during the annotation process.

##### 3.2.1.1. Text span identification

Detailed instructions are given in the annotation guidelines on how to determine the text spans of entities. For example, when annotating an EXAMPLE_LABEL entity, only the actual example index should be annotated, and any word preceding the index, such as “Example,” “Step,” or “Intermediate” should be excluded. In our example snippet Figure 1, “[Step 3]” indicates an example label, but only “3” should be annotated as an EXAMPLE_LABEL entity.

##### 3.2.1.2. Label assignment

Label definitions in Table 2 should be used to choose the correct label for each entity. In addition, more detailed rules have been set out to cover the cases where annotators may have disagreement. These additional rules are especially important since we have the label “OTHER_COMPOUND” which covers all chemical compounds that do not belong to the other four compound labels. Thus, the decision boundaries between the compound labels need to be rigorously defined. One example of such rules is that solvents that are used in work-up procedures should not be annotated as SOLVENT but rather as OTHER_COMPOUND.

#### 3.2.2. Event Annotation Guidelines

A chemical reaction process is usually a sequence of steps, and these steps can be categorized into two types: (1) *reaction steps*, i.e., the steps required to convert the starting materials to the target reaction product; and (2) *work-up steps*, i.e., the manipulations required to purify or isolate a chemical product. For example, in Figure 1, the step of stirring the solution at 80° is a reaction step while the step of concentrating the reaction mixture is a work-up step.

In our corpus, events are quantified by two types of information: the trigger words that flag the occurrences of event steps, and the relations between named entities and trigger words that tell us how entities are involved in event steps. Two labels of trigger words, WORKUP and REACTION_STEP are defined for the two types of event steps, respectively. To capture the relationships between trigger words and named entities, we adapt semantic argument role labels **Arg1** and **ArgM** from the Proposition Bank (Palmer et al., 2005) to label relations. The label Arg1 represents argument roles, a.k.a. thematic roles, being causally affected by another participant in the event (Jurafsky and Martin, 2009), and is therefore used to label the relation between a trigger word and a chemical compound. The label ArgM represents adjunct roles with respect to an event, and thus, is used to label the relation between a trigger word and a temperature, time or yield entity. The definitions of trigger word types and relation types are summarized in Table 2.

### 3.3. Annotation Process

To facilitate the annotation process, a *silver standard* set was first prepared based on information captured in the Elsevier Reaxys® database.^12^ The extracted records from Reaxys are linked to the IDs of their source patents. However, the precise locations of the key entity and relation information in these records in source patents are needed to construct the gold-standard corpus. The silver-standard dataset was prepared by automatically mapping elements of the records in the Reaxys database to the source patents from which the records were extracted. This mapping process was performed by scanning patent texts and searching for excerpted entity mentions.

Three chemical experts were hired to prepare the *gold standard* corpus. They manually reviewed all texts and pre-annotations in the silver-standard dataset to add or correct precise locations of the relevant entities and relations in the texts, according to annotation guidelines in section 3.2.1. Two of the experts first independently reviewed and updated the silver standard annotations. Then, a third chemical expert served as an adjudicator who resolved their disagreements to produce the final gold-standard corpus. See section 3.5 for further details on corpus statistics and data quality.

**Figure 2:**
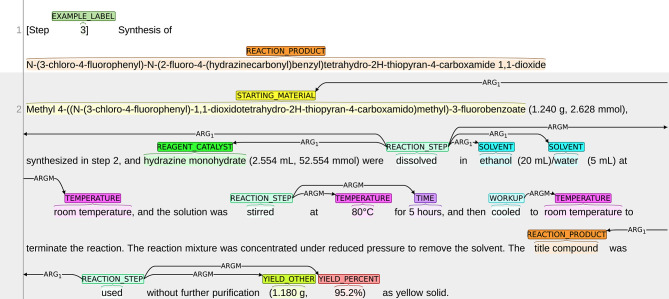
Visualization of the annotations in the snippet in Figure 1.

The annotation process was conducted using the BRAT annotation tool,^13^ which is an interactive web-based tool for adding annotations to input texts. Continuing with the example snippet shown in Figure 1, a visualization of the snippet after annotation is presented in Figure 2. The visualization of our sample dataset, which is a subset of ChEMU chemical reaction corpus and consists of 50 snippets, is available in a dedicated website^14^.

### 3.4. Data Format

The ChEMU chemical reaction corpus is delivered in the BRAT standoff format (Stenetorp et al., 2012). For each snippet, two files were generated by the BRAT annotation tool: a text file (.txt) for the plain text in the snippet, and an annotation file (.ann) consisting of all the annotated entities and events. In the annotation file, each entity/trigger word is represented with a 5-tuple 〈ID, label, *s, e*, text〉. Here, ID represents the index of the annotation, and *s* and *e* represent the offset (position relative to the first character in the snippet) of the starting and ending character of the entity, respectively. Each relation is represented with a 4-tuple: 〈ID, label, Arg1, Arg2〉, where ID represents the index of the annotation, and Arg1 and Arg2 correspond to the IDs of the two entities linked by this relation.

**Figure 3:**
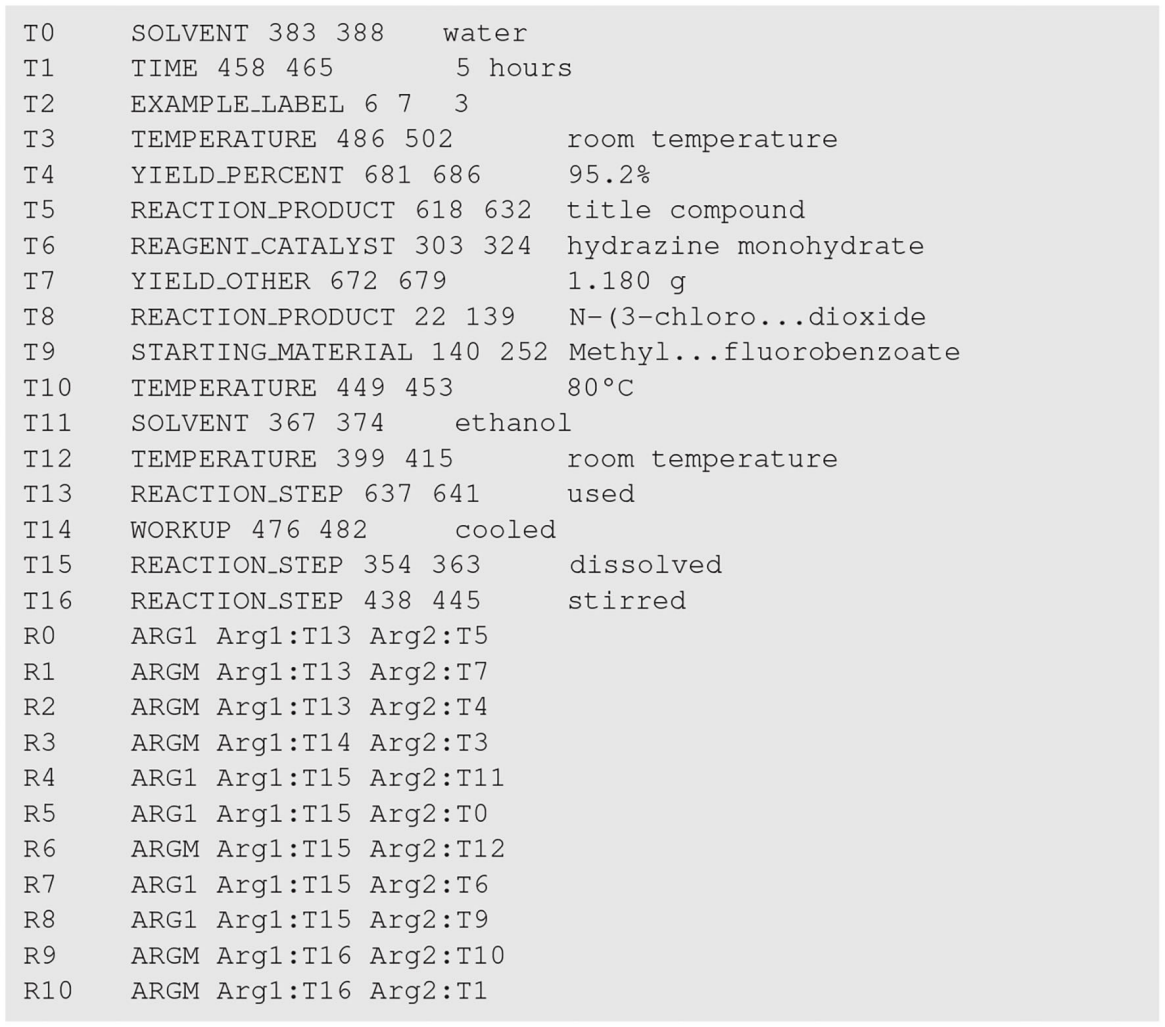
The annotation file for the patent snippet in Figure 1. Entities and trigger words are indexed from T0 to T16, and relations are indexed from R0 to R10.

To give an example, the annotation file for the snippet in Figure 1 is presented in Figure 3. Note that in Figure 3, the texts of the two entities T8 and T9 are abbreviated for ease of presentation.

### 3.5. Annotated Corpus Statistics

#### 3.5.1. Overall Statistics

The overall statistics of our annotated corpus are presented in Table 3. In the 1,500 selected patent snippets, 26,857 entities and 11,236 trigger words were annotated, with 23,445 relations identified between them. The numbers of instances annotated for each label defined in Table 4 are summarized in Table 2.

**Table 3:** Overall statistics of the annotated corpus.

**Feature**	**Value**
# Patent snippets	1,500
# Total entities	26,857
# Trigger words	11,236
# Relations	23,445

**Table 4:** Number of instances for each label defined in Table 2.

**Label**	**# Instances**
STARTING_MATERIAL	2,878
REAGENT_CATALYST	2,074
REACTION_PRODUCT	3,413
SOLVENT	1,818
OTHER_COMPOUND	7,651
TIME	1,763
TEMPERATURE	2,473
YIELD_PERCENT	1,572
YIELD_OTHER	1,762
EXAMPLE_LABEL	1,453
REACTION_STEP	6,210
WORKUP	5,026
Arg1	15,865
ArgM	7,580

#### 3.5.2. Inter-Annotator Agreement

We measure the inter-annotator agreement (IAA) of our corpus using two metrics: (1) Cohen's Kappa (Cohen, 1960) and (2) F_1_-scores. Cohen's Kappa is a standard metric for the evaluation of inter-annotator agreement. But for the tasks in ChEMU 2020 lab, i.e., named entity recognition (NER) and event extraction (EE), Cohen's Kappa score may not the best metric to quantify the extent of agreement between annotators (Hripcsak and Rothschild, 2005; Grouin et al., 2011). This is because the computation of Kappa requires the number of negative cases to be known, which is not explicitly given in the tasks of NER or EE. Thus, the pairwise F_1_-scores are also commonly used to measure the IAA scores. In this paper, we report the IAA scores using both metrics. In the computation of Cohen's Kappa scores, we only consider the set of entities that are annotated by at least one annotator. The IAA scores in both metrics are summarized in Table 5. Note that in this table, “anno3” represents the third annotator (the adjudicator) as discussed in section 3.3 and thus, the annotations by “anno3” are the same as the annotations in gold-standard corpus.

**Table 5:** Summary of inter-annotator agreement scores.

**Metric**	**anno1 vs. anno2**	**anno1 vs. anno3**	**anno2 vs. anno3**
**Named entities**			
Cohen's Kappa	0.9070	0.9515	0.9539
F_1_ score	0.9505	0.9747	0.9760
**Events**			
Cohen's Kappa	0.6513	0.8035	0.8068
F_1_ score	0.8985	0.9496	0.9506

### 3.6. Data Partitions

The ChEMU chemical reaction corpus was randomly partitioned into train/development/test splits with the ratio of 0.6/0.15/0.25. The training and development sets were released to participants for model development and the test set was withheld for use in the evaluation stage.

**Figure 4:**
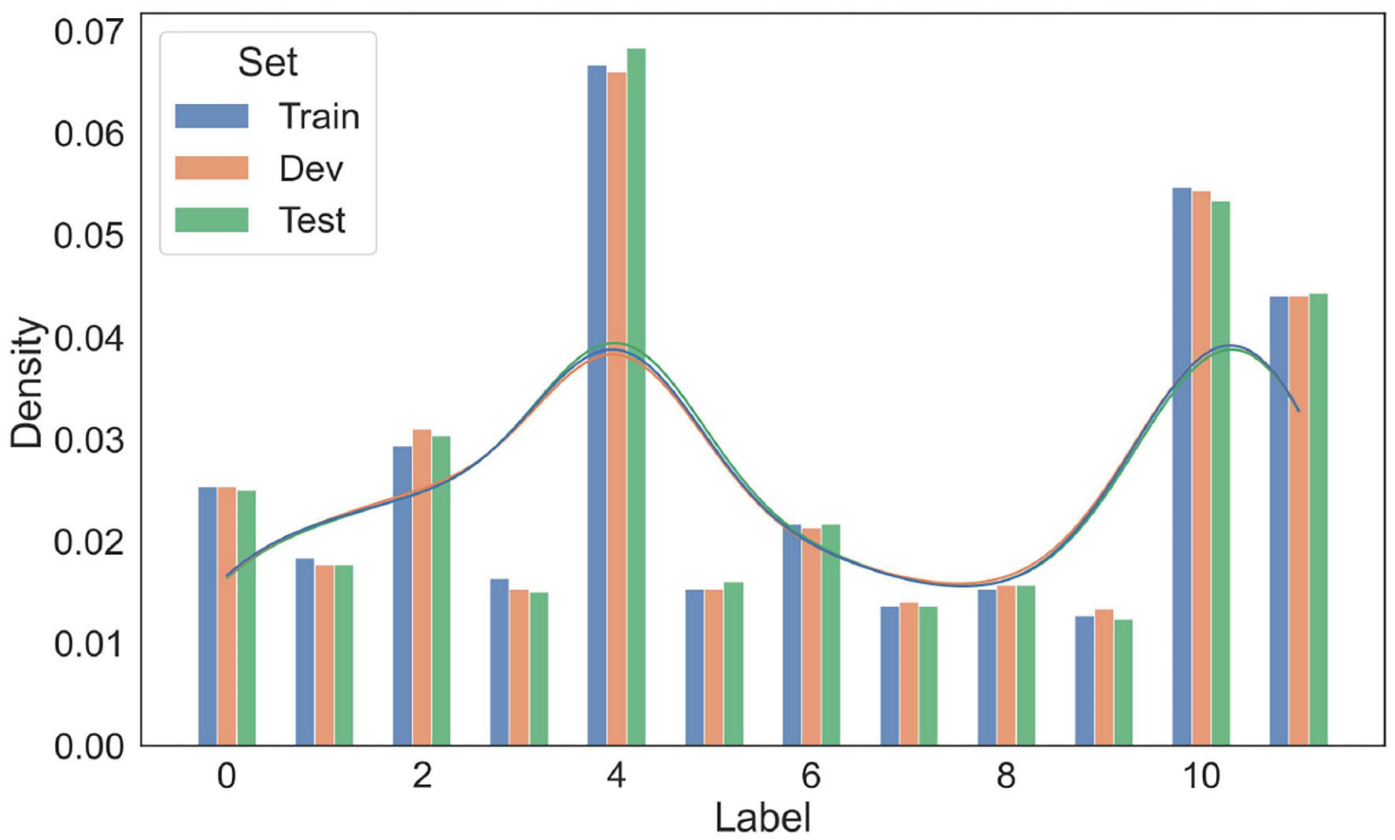
Distributions of entity labels on the training/development/test data splits. The labels are indexed according to their order in Table 4.

To ensure a fair split of data, two statistical tests on the resultant train/dev/test splits were conducted. The distributions of entity labels (10 entity labels plus two trigger word labels in Table 2) in train/dev/test sets are presented in Figure 4. As shown in this figure, the distributions over entity labels on train/dev/test sets are quite similar. Specifically, only slight fluctuations in label distributions (≤0.004) are found across the three splits.

**Figure 5:**
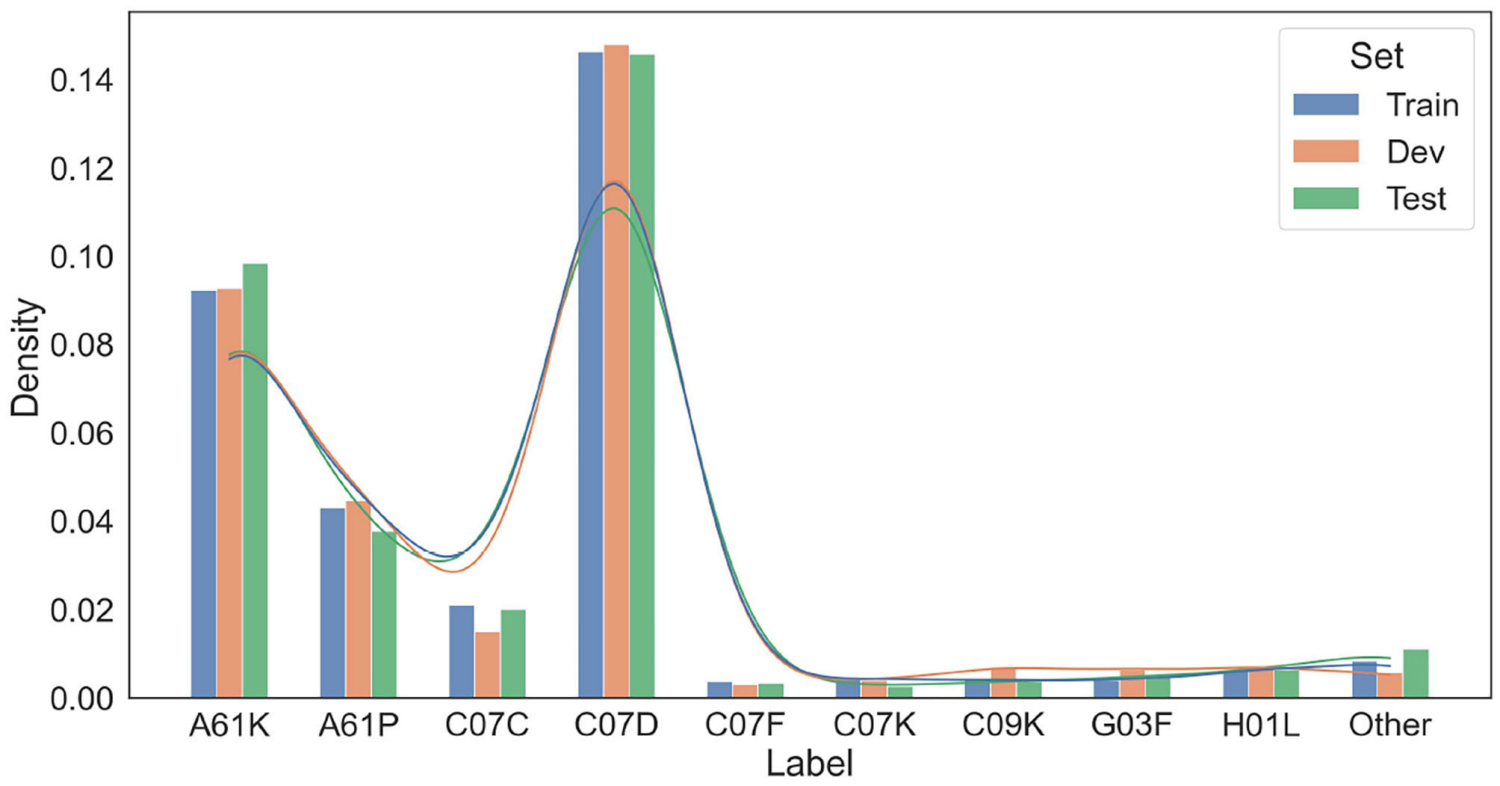
Distributions of IPCs on the training/development/test data splits. Only dominating IPC groups that take up more than 1% of at least one data split are included in this figure. Other IPCs are grouped as “Other”.

We further compare the train/dev/test splits in terms of the International Patent Classifications (IPCs)^15^ of their source patents. The IPC information of a patent reflects its application category. For example, the IPC code “A61K” is assigned to patents for preparations for medical, dental, or toilet purposes. Since patents with different IPCs may differ in the vocabulary and linguistic properties, we want to make sure that the patent snippets in the train, dev, and test set have similar distributions over IPCs. For each patent snippet, we extract the primary IPC of its corresponding source patent, and summarize the IPC distributions of the snippets in train/dev/test sets in Figure 5. As shown in Figure 5, the IPC distributions across the three splits are also similar.

## 4. The Tasks

We provided two tasks in ChEMU lab: Task 1—Named Entity Recognition (NER), and Task 2—Event Extraction (EE). We also hosted a third track where participants can work on development of end-to-end systems which address both tasks jointly.

### 4.1. Overview of Tasks

#### 4.1.1. Task 1: Named Entity Recognition (NER)

The first task aims to identify named entities that occur in the descriptions of chemical reactions. The task requires (1) detection of text spans of named entities and (2) assigning the correct labels to detected entities from the set of labels defined in Table 2. For example, in Figure 1, the entity “hydrazine monohydrate” (line 4) needs to be detected and assigned with the label REACTION_CATALYST, according to its role in the chemical reaction.

#### 4.1.2. Task 2: Event Extraction (EE)

A chemical reaction usually consists of an ordered sequence of event steps that transforms a starting material into an end product, such as the five reaction steps for the example snippet in Figure 1. The event extraction task (Task 2) targets identifying these event steps.

Similar with conventional event extraction problems (Kim et al., 2009), Task 2 involves three subtasks: (1) trigger word prediction and (event) typing; (2) argument prediction; and (3) semantic role typing. First, it requires the identification of trigger words. For each trigger word detected, one label out of the trigger labels defined in Table 2 needs to be assigned. Second, it requires the determination of argument entities that are associated with the trigger words, i.e., which entities identified in Task 1 participate in event or reaction steps. This is done by labeling the connections between event trigger words and their arguments. Finally, Task 2 requires the assignment of correct role types (Arg1 or ArgM) to each of the detected relations.

#### 4.1.3. Task 3: End-to-End Systems

The third track allows participants to develop end-to-end systems that address both tasks simultaneously, i.e., the extraction of reaction events including their constituent entities directly from chemical patent snippets. This is a more realistic scenario for an event extraction system to be applied for large-scale annotation of events.

#### 4.1.4. Workflow of the Three Tracks

**Figure 6:**
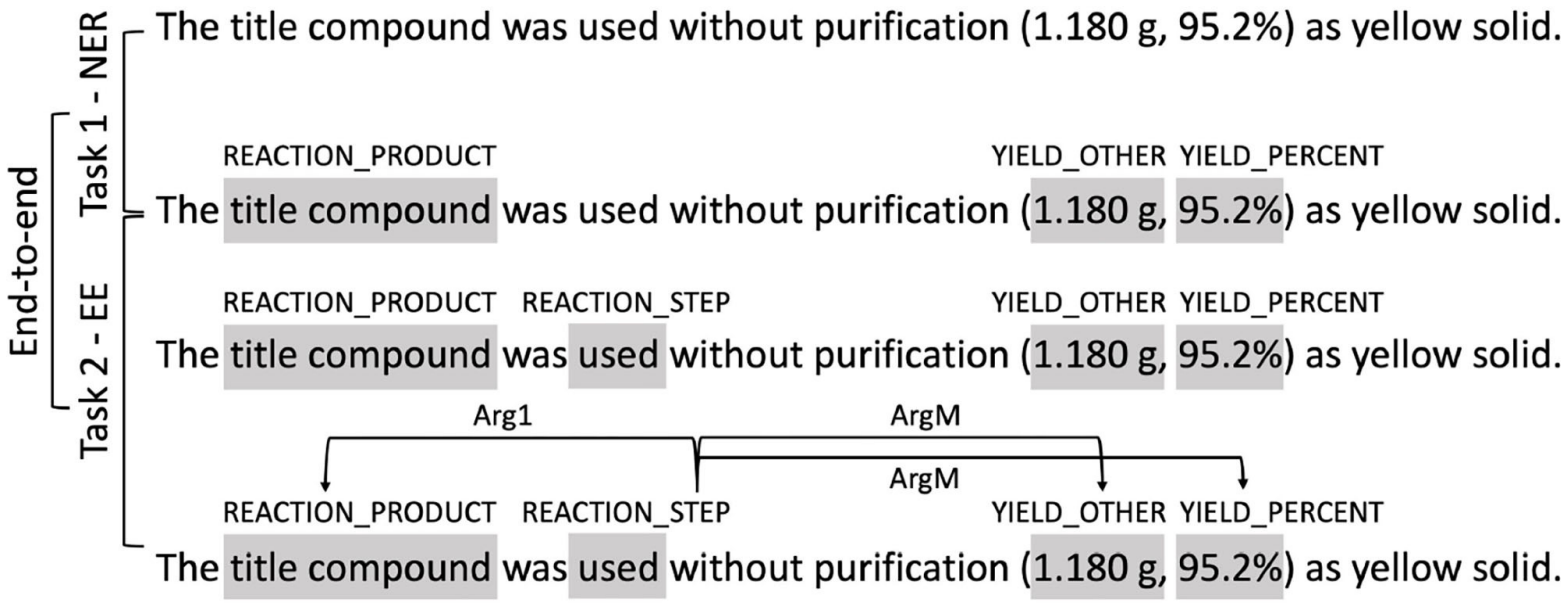
Illustration of the three tasks. Shaded text spans represent annotated entities or trigger words. Arrows represent relations between entities.

The workflows of the three tracks is illustrated in Figure 6, where the input and output of each track is illustrated using a the last sentence in Figure 1 as an example. The input of Task 1 NER is the plain text of the snippet. Participants need to identify entities defined in Table 2, e.g., the text span “title compound” need to be identified as “REACTION_PRODUCT.” The input of Task 2 EE is the plain text plus the ground-truth named entities. Participants are required to firstly identify the trigger words and their types (e.g., the text span “used” is identified as “REACTION_STEP”) and then identify the relations between the trigger words and the provided entities (e.g., a directed link from “used” to “title compound” is added and labeled as “Arg1”). In the track of end-to-end systems, participants are only provided with the plain text. They are required to identify both the entities and the trigger words, and predict the event steps directly from the text.

### 4.2. Organization of Tracks

#### 4.2.1. Training Stage

The training and development data sets were released to all participants for model development. Two different versions of training data, namely Data-NER and Data-EE, were provided. Data-NER was prepared for participants in Task 1, where the gold-standard entities were included. Data-EE was prepared for Tasks 2 and 3, where both the gold-standard entities, trigger words and entity relations were included.

#### 4.2.2. Testing Stage

Since the gold-standard entities of the test set needed to be provided to participants in Task 2, the testing stage of Task 2 was delayed until after the testing of Tasks 1 and 3 are completed, so as to prevent data leakage.

Therefore, our testing stage consists of two phases. In the first phase, the text (.txt) files of all test snippets were released. Participants in Task 1 are required to use the released patent texts to predict the entities. Participants in Task 3 were required to also predict the trigger words and entity relations.

In the second phase, the gold-standard entities of all test snippets were released. Participants in Task 2 can use the released gold-standard entities, along with the text files released in the first phase, to predict the event steps in test snippets.

#### 4.2.3. Submission Website

A submission website was developed and maintained, which allows participants to submit their runs during the testing stage.^16^ In addition, the website offers several other important functions to facilitate organization of the lab.

First, it hosts the download links for the training, development, and test data sets so that participants can access the data sets conveniently. Second, it allows participants to test the performance (against the development set) of their models before the testing stage starts, which also offers a chance for participants to familiarize themselves with the evaluation tool BRATEval^17^ (detailed in section 5). The website also hosts a private leaderboard for each team that ranks all runs submitted by each team, and a public leaderboard that ranks all runs that have been made public by teams.

#### 4.2.4. Timeline

The timeline of each stage is summarized as follows.

- Release of sample data: 09 March 2020
- Release of training data: 10 April 2020
- Testing stage (phase 1): 22 May 2020–28 May 2020
- Testing stage (phase 2): 29 May 2020–3 June 2020
- End of evaluation cycle and feedback to participants: 05 June 2020

## 5. Evaluation Framework

In this section, we describe the evaluation framework of the ChEMU lab. We introduce three baseline algorithms for Task 1, Task 2, and end-to-end systems, respectively.

### 5.1. Evaluation Methods

We use BRATEval to evaluate all the runs that we receive. Three metrics are used to evaluate the performance of all the submissions for Task 1: Precision, Recall, and F_1_-score. Specifically, given a predicted entity and a ground-truth entity, we treat the two entities as a match if (1) the types associated with the two entities match; and (2) their text spans match. The overall Precision, Recall, and F_1_-score are computed by micro-averaging all instances (entities).

In addition, we exploit two different matching criteria, exact-match and relaxed-match, when comparing the texts spans of two entities. Here, the exact-match criterion means that we consider that the text span of an entity matches with that of another entity if both the starting and the end offsets of their spans match. The relaxed-match criterion means that we consider that the text span of one entity matches with that of another entity as long as their text spans overlap.

The submissions for Task 2 and end-to-end systems are evaluated using Precision, Recall, and F_1_-score by comparing the predicted events and gold standard events. We consider two events as a match if (1) their trigger words, event types and semantic roles are the same; and (2) the entities involved in the two events match. Here, we follow the method in Task 1 to test whether two entities match. This means that the matching criteria of exact-match and relaxed-match are also applied in the evaluation of Task 2 and of end-to-end systems. Note that the relaxed-match will only be applied when matching the spans of two entities; it does not relax the requirement that the entity type of predicted and ground truth entities must agree. Since Task 2 provides gold entities but not event triggers with their ground truth spans, the relaxed-match only reflects the accuracy of spans of predicted trigger words.

**Figure 7:**
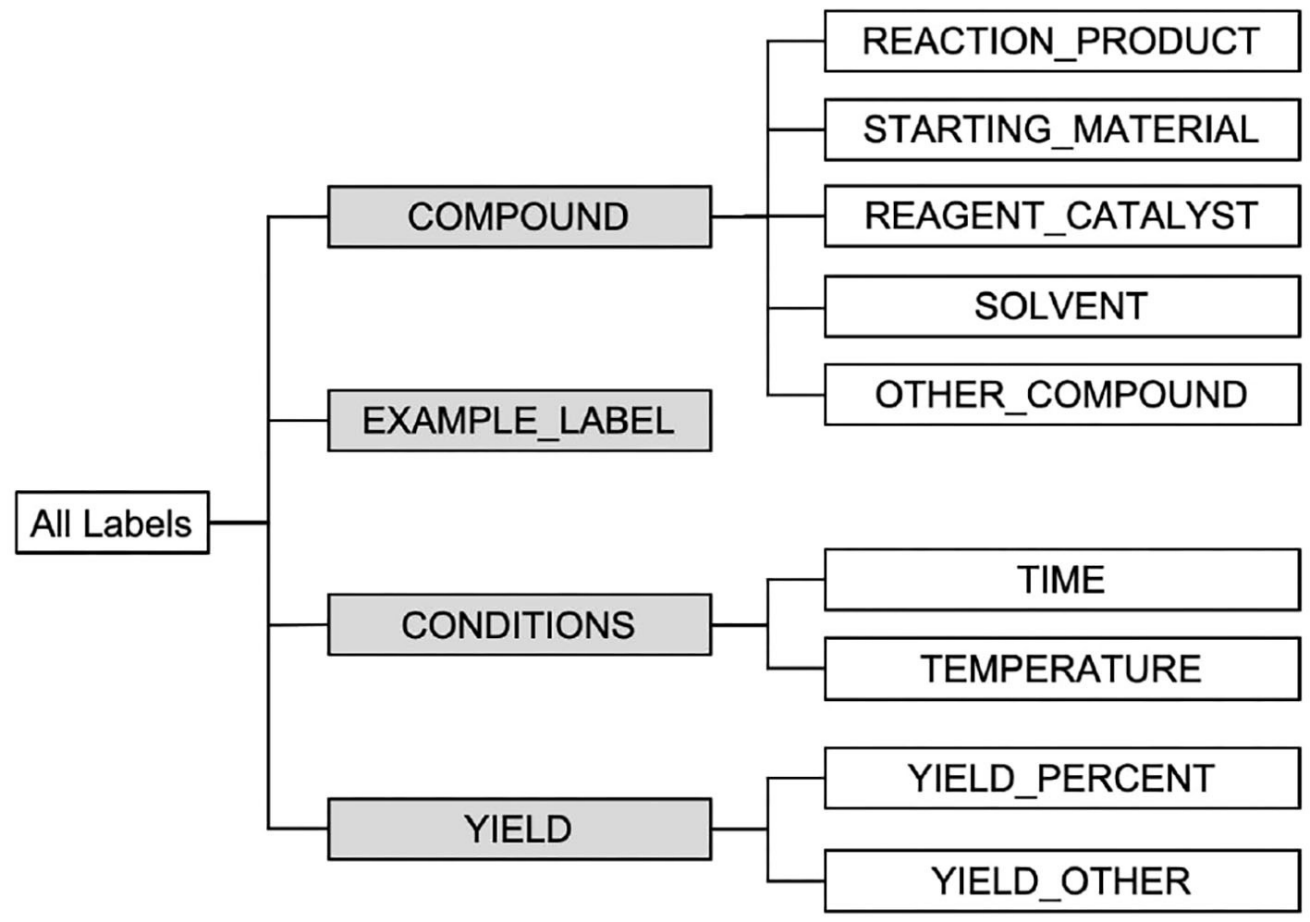
Illustration of the hierarchical NER class structure used in evaluation.

To somewhat accommodate a relaxed form of entity type matching, we also evaluate submissions in Task 1—NER using a set of high-level labels shown in the hierarchical structure of entity classes in Figure 7. The higher-level labels used are highlighted in gray. In this set of evaluations, given a predicted entity and a ground-truth entity, we consider that their labels match as long as their corresponding high-level labels match. For example, suppose we get as predicted entity “STARTING_MATERIAL, [335, 351), boron tribromide” while the (correct) ground-truth entity instead reads “REAGENT_CATALYST, [335, 351), boron tribromide,” where each entity is presented in the form of “TYPE, SPAN, TEXT.” In the evaluation framework described earlier this example will be counted as a mismatch. However, in this additional set of entity type relaxed evaluations we consider the two entities as a match, since both labels “STARTING_MATERIAL” and “REAGENT_CATALYST” specialize their parent label “COMPOUND.”

### 5.2. Baselines

We released one baseline method for each task as a benchmark method. Specifically, the baseline for Task 1 is based on retraining **BANNER** (Leaman and Gonzalez, 2008) on the training and development data; the baseline for Task 2 is a co-occurrence method; and the baseline for end-to-end systems is a two-stage algorithm that first uses BANNER to identify entities in the input and then uses the co-occurrence method to extract events.

#### 5.2.1. BANNER

BANNER is a named entity recognition tool for biomedical data. In this baseline, we first use the GENIA Sentence Splitter (GeniaSS) (Sætre et al., 2007) to split input texts into separate sentences. The resulting sentences are then fed into BANNER, which predicts the named entities using three steps, namely tokenization, feature generation, and entity labeling. A simple tokenizer is used to break sentences into either a contiguous block of letters and/or digits or a single punctuation mark. BANNER uses a conditional random field (CRF) implementation derived from the MALLET toolkit^18^ for feature generation and token labeling. The set of machine learning features used consist primarily of orthographic, morphological and shallow syntax features.

#### 5.2.2. Co-occurrence Method

This method first creates a dictionary *D*_*e*_ for the observed trigger words and their corresponding types from the training and development sets. For example, if a word “added” is annotated as a trigger word with the label of “WORKUP” in the training set, we add an entry 〈added, WORKUP〉 to *D*_*e*_. In the case where the same word has been observed to appear as both types of “WORKUP” and “REACTION_STEP,” we only keep as entry in *D* its most frequent label. The method also creates an event dictionary *D*_*r*_ for the observed semantic roles in the training and development sets. For example, if an event 〈ARG1, E1, E2〉 is observed where “E1” corresponds to trigger word “added” of type “WORKUP” and “E2” corresponds to entity “water” of type “OTHER_COMPOUND,” we add an entry 〈ARG1, WORKUP, OTHER_COMPOUND〉 to *D*_*r*_.

To predict events, this method first identifies all trigger words in the test set using *D*_*e*_. It then extracts two events 〈ARG1, T1, T2〉 and 〈ARGM, T1, T2〉 for a trigger word “E1” and an entity “E2” if (1) they co-occur in the same sentence; and (2) the relation type 〈ARGx, T1, T2〉 is included in *D*_*r*_. Here, “ARGx” can be “ARG1” or “ARGM,” and “T1” and “T2” are the entity types of “E1” and “E2,” respectively.

#### 5.2.3. BANNER + Co-occurrence Method

The above two baselines are combined to form a two-stage method for end-to-end systems. This baseline first uses BANNER to identify all the entities in Task 1. Then it utilizes the co-occurrence method to predict events, except that gold standard entities are replaced with the entities predicted by BANNER in the first stage.

## 6. Results

In total, 39 teams registered for the ChEMU shared task, of which 36 teams registered for Task 1, 31 teams registered for Task 2, and 28 teams registered for both tasks. The 39 teams are spread across 13 different countries, from both the academic and industry research communities. In this section, we report the results of all the runs that we received for each task.

### 6.1. Task 1—Named Entity Recognition

Task 1 received considerable interest with the submission of 25 runs from 11 teams. The 11 teams include one team from Germany (OntoChem), three teams from India (AUKBC, SSN_NLP, and JU_INDIA), one team from Switzerland (BiTeM), one team from Portugal (Lasige_BioTM), one team from Russia (KFU_NLP), one team from the United Kingdom (NextMove Software/Minesoft), two teams from the United States of America (Melaxtech and NLP@VCU), and one team from Vietnam (VinAI). We evaluate the performance of all 25 runs, comparing their predicted entities with the ground-truth entities of the patent snippets in the test set. We report the performances of all runs under both matching criteria in terms of three metrics, namely Precision, Recall, and F_1_-score.

We report the overall performance of all runs in Table 6. The baseline of Task 1 achieves 0.8893 in F_1_-score under exact match. Nine runs outperform the baseline in terms of F_1_-score under exact match. The best run was submitted by team Melaxtech, achieving a high F_1_-score of 0.9570. There were sixteen runs with an F_1_-score >0.90 under relaxed-match. However, under exact-match, only seven runs surpassed 0.90 in F_1_-score. This difference between exact-match and relaxed-match may be related to the long text spans of chemical compounds, which is one of the main challenges in NER tasks in the domain of chemical documents.

**Table 6:** Overall performance of all runs in Task 1—Named entity recognition.

**Run**	**Exact-match**			**Relaxed-match**		
	**P**	**R**	**F**	**P**	**R**	**F**
Melaxtech-run1	0.9571	**0.9570**	**0.9570**	0.9690	*0.9687*	*0.9688*
Melaxtech-run2	**0.9587**	*0.9529*	*0.9558*	0.9697	0.9637	0.9667
Melaxtech-run3	*0.9572*	0.9510	0.9541	0.9688	0.9624	0.9656
VinAI-run2^*^	0.9538	0.9504	0.9521	**0.9737**	**0.9716**	**0.9726**
VinAI-run1	0.9462	0.9405	0.9433	*0.9707*	0.9661	0.9684
Lasige_BioTM-run1	0.9327	0.9457	0.9392	0.9590	0.9671	0.9630
BiTeM-run3	0.9378	0.9087	0.9230	0.9692	0.9558	0.9624
BiTeM-run2	0.9083	0.9114	0.9098	0.9510	0.9684	0.9596
NextMove/Minesoft-run1	0.9042	0.8924	0.8983	0.9301	0.9181	0.9240
NextMove/Minesoft-run2	0.9037	0.8918	0.8977	0.9294	0.9178	0.9236
**Baseline**	0.9071	0.8723	0.8893	0.9219	0.8893	0.9053
NLP@VCU-run1	0.8747	0.8570	0.8658	0.9524	0.9513	0.9518
KFU_NLP-run1	0.8930	0.8386	0.8649	0.9701	0.9255	0.9473
NLP@VCU-run2	0.8705	0.8502	0.8602	0.9490	0.9446	0.9468
NLP@VCU-run3	0.8665	0.8514	0.8589	0.9486	0.9528	0.9507
KFU_NLP-run2	0.8579	0.8329	0.8452	0.9690	0.9395	0.9540
NextMove/Minesoft-run3	0.8281	0.8083	0.8181	0.8543	0.8350	0.8445
KFU_NLP-run3	0.8197	0.8027	0.8111	0.9579	0.9350	0.9463
BiTeM-run1	0.8330	0.7799	0.8056	0.8882	0.8492	0.8683
OntoChem-run1	0.7927	0.5983	0.6819	0.8441	0.6364	0.7257
AUKBC-run1	0.6763	0.4074	0.5085	0.8793	0.5334	0.6640
AUKBC-run2	0.4895	0.1913	0.2751	0.6686	0.2619	0.3764
SSN_NLP-run1	0.2923	0.1911	0.2311	0.8633	0.4930	0.6276
SSN_NLP-run2	0.2908	0.1911	0.2307	0.8595	0.4932	0.6267
JU_INDIA-run1	0.1411	0.0824	0.1041	0.2522	0.1470	0.1857
JU_INDIA-run2	0.0322	0.0151	0.0206	0.1513	0.0710	0.0966
JU_INDIA-run3	0.0322	0.0151	0.0206	0.1513	0.0710	0.0966

*Here, P, R, and F represents the Precision, Recall, and F_1_-score, respectively. For each metric, we highlight the best result in **bold** and the second best result in italic. The results are ordered by their performance in terms of F_1_-score under exact-match*.

* *This run was received after evaluation phase and thus was not included in official results*.

Next, we evaluate the performance of all 25 runs using the high-level labels in Figure 7 (highlighted in gray). We report the performances of all runs in terms of Precision, Recall, and F_1_-score in Table 7.

**Table 7:** Overall performance of all runs in Task 1—Named entity recognition where the set of high-level labels in Figure 7 is used.

**Run**	**Exact-match**			**Relaxed-match**		
	**P**	**R**	**F**	**P**	**R**	**F**
Melaxtech-run1	*0.9774*	**0.9774**	**0.9774**	0.9906	0.9901	0.9903
Melaxtech-run2	**0.9789**	*0.9732*	*0.9760*	0.9910	0.9849	0.9879
Melaxtech-run3	0.9775	0.9714	0.9744	0.9905	0.9838	0.9871
VinAI-run2^*^	0.9704	0.9670	0.9687	**0.9920**	0.9901	*0.9911*
Lasige_BioTM-run1	0.9571	0.9706	0.9638	0.9886	*0.9943*	**0.9915**
VinAI-run1	0.9635	0.9579	0.9607	0.9899	0.9854	0.9877
**Baseline**	0.9657	0.9288	0.9469	0.9861	0.9519	0.9687
BiTeM-run1	0.9573	0.9277	0.9423	0.9907	0.9770	0.9838
NextMove/Minesoft-run2	0.9460	0.9330	0.9394	0.9773	0.9611	0.9691
NextMove/Minesoft-run1	0.9458	0.9330	0.9393	0.9773	0.9610	0.9691
BiTeM-run2	0.9323	0.9357	0.9340	0.9845	**0.9962**	*0.9903*
NextMove/Minesoft-run3	0.9201	0.8970	0.9084	0.9571	0.9308	0.9438
NLP@VCU-run1	0.9016	0.8835	0.8925	0.9855	0.9814	0.9834
NLP@VCU-run2	0.9007	0.8799	0.8902	0.9882	0.9798	0.9840
NLP@VCU-run3	0.8960	0.8805	0.8882	0.9858	0.9869	0.9863
KFU_NLP-run1	0.9125	0.8570	0.8839	*0.9911*	0.9465	0.9683
BiTeM-run3	0.9073	0.8496	0.8775	0.9894	0.9355	0.9617
KFU_NLP-run2	0.8735	0.8481	0.8606	0.988	0.9569	0.9722
KFU_NLP-run3	0.8332	0.8160	0.8245	0.9789	0.9516	0.9651
OntoChem-run1	0.9029	0.6796	0.7755	0.9611	0.7226	0.8249
AUKBC-run1	0.7542	0.4544	0.5671	0.9833	0.5977	0.7435
AUKBC-run2	0.6605	0.2581	0.3712	0.9290	0.3612	0.5201
SSN_NLP-run2	0.3174	0.2084	0.2516	0.9491	0.5324	0.6822
SSN_NLP-run1	0.3179	0.2076	0.2512	0.9505	0.5304	0.6808
JU_INDIA-run1	0.2019	0.1180	0.1489	0.5790	0.3228	0.4145
JU_INDIA-run2	0.0557	0.0262	0.0357	0.4780	0.2149	0.2965
JU_INDIA-run3	0.0557	0.0262	0.0357	0.4780	0.2149	0.2965

*Here, P, R, and F represents the Precision, Recall, and F_1_-score, respectively. For each metric, we highlight the best result in **bold** and the second best result in italic. The results are ordered by their performance in terms of F_1_-score under exact-match*.

* *This run was received after evaluation phase and thus was not included in official results*.

### 6.2. Task 2—Event Extraction

We received ten (10) runs from five (5) teams. Specifically, the five teams include one team from Portugal (Lasige_BioTM), one team from Turkey (BOUN_REX), one team from the United Kingdom (NextMove Software/Minesoft) and two teams from the United States of America (Melaxtech and NLP@VCU). We evaluate all runs using the metrics Precision, Recall, and F_1_-score. Again, we utilize the two matching criteria, namely exact-match and relaxed-match, when comparing the trigger words in the submitted runs and ground-truth data.

The overall performance of each run is summarized in Table 8.^19^ The baseline (co-occurrence method) scored relatively high in Recall, i.e, 0.8861. This was expected, since the co-occurrence method aggressively extracts all possible events within a sentence. However, the F_1_-score was low due to its low Precision score. Here, all runs outperform the baseline in terms of F_1_-score under exact-match. Melaxtech ranks first among all official runs in this task, with an F_1_-score of 0.9536.

**Table 8:** Overall performance of all runs in Task 2—Event extraction.

**Run**	**Exact-match**			**Relaxed-match**		
	**P**	**R**	**F**	**P**	**R**	**F**
Melaxtech-run1	*0.9568*	**0.9504**	**0.9536**	*0.9580*	**0.9516**	**0.9548**
Melaxtech-run2	**0.9619**	0.9402	*0.9509*	**0.9632**	0.9414	*0.9522*
Melaxtech-run3	0.9522	*0.9437*	0.9479	0.9534	*0.9449*	0.9491
NextMove/Minesoft-run1	0.9441	0.8556	0.8977	0.9441	0.8556	0.8977
NextMove/Minesoft-run2	0.8746	0.7816	0.8255	0.8909	0.7983	0.8420
BOUN_REX-run1	0.7610	0.6893	0.7234	0.7610	0.6893	0.7234
NLP@VCU-run1	0.8056	0.5449	0.6501	0.8059	0.5451	0.6503
NLP@VCU-run2	0.5120	0.7153	0.5968	0.5125	0.7160	0.5974
NLP@VCU-run3	0.5085	0.7126	0.5935	0.5090	0.7133	0.5941
**Baseline**	0.2431	0.8861	0.3815	0.2431	0.8863	0.3816

*Here, P, R, and F represent the Precision, Recall, and F_1_-score, respectively. For each metric, we highlight the best result in **bold** and the second best result in italics. The results are ordered by their performance in terms of F_1_-score under exact-match*.

### 6.3. End-to-End Systems

We received 10 end-to-end system runs from four teams. The four teams include one team from Germany (OntoChem), one team from Portugal (Lasige_BioTM), one team from the United Kingdom (NextMove Software/Minesoft), and one team from the United States of America (Melaxtech).

The overall performance of all runs is summarized in Table 9 in terms of Precision, Recall, and F_1_-score under both exact-match and relaxed-match.^20^ Since gold entities are not provided in this task, the average performance of the runs in this task are slightly lower than those in Task 2. Note that the Recall scores of most runs are substantially lower than their Precision scores. This may reveal that the task of identifying a relation from a chemical patent is harder than the task of typing an identified relation. The first run from Melaxtech team ranks best among all runs received for this task.

**Table 9:** Overall performance of all runs in end-to-end systems.

**Run**	**Exact-match**			**Relaxed-match**		
	**P**	**R**	**F**	**P**	**R**	**F**
Melaxtech-run1	**0.9201**	**0.9147**	**0.9174**	**0.9319**	**0.9261**	**0.9290**
NextMove/Minesoft-run1	*0.8492*	*0.7609*	*0.8026*	*0.8663*	*0.7777*	*0.8196*
NextMove/Minesoft-run2	0.8486	0.7602	0.8020	0.8653	0.7771	0.8188
NextMove/Minesoft-run3	0.8061	0.7207	0.7610	0.8228	0.7371	0.7776
OntoChem-run1	0.7971	0.3777	0.5126	0.8407	0.3984	0.5406
OntoChem-run2	0.7971	0.3777	0.5126	0.8407	0.3984	0.5406
OntoChem-run3	0.7971	0.3777	0.5126	0.8407	0.3984	0.5406
**Baseline**	0.2104	0.7329	0.3270	0.2135	0.7445	0.3319
Melaxtech-run2	0.2394	0.2647	0.2514	0.2429	0.2687	0.2552
Melaxtech-run3	0.2383	0.2642	0.2506	0.2421	0.2684	0.2545

*Here, P, R, and F represent the Precision, Recall, and F_1_-score, respectively. For each metric, we highlight the best result in bold and the second best result in italics. The results are ordered by their performance in terms of F_1_-score under exact-match*.

## 7. Participants' Approaches

We received paper submissions from eight teams include team BiTeM, VinAI, BOUN-REX, NextMove/Minesoft, NLP@VCU, AU-KBC, LasigBioTM, and Melaxtech. The tasks that the eight teams have participated in are summarized in Table 10. In this section, we compare and summarize how the eight teams address these tasks. More details are available in the CLEF2020 workshop papers (He et al., 2020b).

**Table 10:** The task participation of the 8 teams BiTeM, VinAI, BOUN-REX, NextMove/Minesoft, NLP@VCU, AU-KBC, LasigBioTM, and Melaxtech.

**Team**	**Task 1**	**Task 2**	**Task 3**
BiTeM	✓		
VinAI	✓		
BOUN-REX		✓	
NextMove/Minesoft	✓	✓	✓
NLP@VCU	✓	✓	
AU-KBC	✓		
LasigBioTM	✓	✓	✓
Melaxtech	✓	✓	✓

### 7.1. BiTeM (Copara et al., 2020b)

The BiTeM team participated in Task 1. They explored various popular structures including Bidirectional Encoder Representations from Transformers (BERT) (Devlin et al., 2019) and its variants, such as ChemBERTa model,^21^ and Convolutional Neural Network (CNN) (Lecun, 1989). Their best system is an ensemble created using a majority vote strategy (Copara et al., 2020a) of three models: the BERT-base-cased model, BERT-base-uncased model, and a CNN model. The BiTeM submitted three runs to Task 1, and their best run was ranked the 7-th among all 26 runs.

### 7.2. VinAI (Dao and Nguyen, 2020)

The VinAI team participated in Task 1. They addressed this task utilizing the BiLSTM-CNN-CRF model (Ma and Hovy, 2016) and further leveraged the ChELMo word embeddings released by Zhai et al. (2019). ChELMo is a contextualized ELMo language model trained from scratch with a corpus of 84K chemical patents. A Word2vec skip-gram model trained on the same corpus being used in the input layer of ChELMo.^22^ The VinAI team submitted two runs to Task 1 including one post-evaluation run, and their post-evaluation run was ranked the 4-th among all 26 runs.

### 7.3. BOUN-REX (Köksal et al., 2020)

The BOUN-REX team participated in Task 2. The team broke the task into two steps: (1) trigger word detection; and (2) determination of trigger type/event type (The event type is determined by trigger type). The team modeled the first step as a question-answering problem, which aims to find the starting and ending index of trigger word spans. The second step was modeled as a classification problem. BERT structures were used to convert input texts into deep latent features, and an objective function that jointly considers the loss w.r.t. the two steps was used in fine-tuning. Various BERT architectures were explored, including BioBERT, BERT-large-uncased, and BERT-large-cased. The BOUN-REX team submitted one run to Task 2, which was ranked the 6-th among all 11 runs.

### 7.4. NextMove Software/Minesoft (Lowe and Mayfield, 2020)

The NextMove Software/Minesoft team participated in all three tasks. They employed the open source tool ChemicalTagger (Hawizy et al., 2011) to detect chemical reactions from patent texts, which directly provides information on chemical entities, chemical properties, trigger words, and Part-of-Speech (PoS) information within input texts. They further adapted ChemicalTagger for tasks in ChEMU lab 2020 in several ways : (1) LeadMine (Lowe and Sayle, 2015) was used to recognized chemicals, i.e., ChemicalTagger's tokenization was adjusted accordingly such that all LeadMine entities were treated as single tokens; and (2) Rules were used to cover the differences between the annotation guidelines of ChEMU and outputs of ChemicalTagger, e.g., the definitions of “catalyst.” The NextMove Software/Minesoft team submitted three runs, two runs, and three runs to Tasks 1–3, respectively. Their best run for each task was ranked the 8-th, 4-th, and 2-nd in Tasks 1–3, respectively.

### 7.5. NLP@VCU (Mahendran et al., 2020)

The NLP@VCU team participated in two tasks: Task 1 and Task 2. The team utilized a BiLSTM-CRF model in Task 1, with a concatenated input of pre-trained word embeddings (Mikolov et al., 2013) and character embeddings. In Task 2, the team proposed two models to identify relations between the trigger words and the entities. Their first approach is a rule-based method, which connects a named entity with its closest trigger word within the same sentence. Their second approach is a CNN-based model, which performs a binary classification to determine if a given trigger-entity pair is related or not. The NLP@VCU team submitted three runs to Task 1 and 2, respectively. Their best run for Task 1 was ranked the 11-th among all 26 runs, and their best run for Task 2 was ranked the 7-th among all 11 runs.

### 7.6. AU-KBC (Pattabhi et al., 2020)

The AU-KBC team participated in Task 1. They investigated two different model architectures to address this task: (1) a CRF model; and (2) an Multi-Layer Perceptron (MLP) model. The inputs of their systems are the syntactic features extracted from patent texts, such as PoS and Phrase Chunk information (noun phrase, verb phrase). Then a CRF model and an MLP model were used to learn to map these input features to desired target outputs, which resulted in the two runs submitted by AU-KBC. They have shown that the CRF model is more effective in terms of capturing contextual information in the inputs, compared with the MLP model. The AU-KBC team submitted two runs to Task 1, and their better run was ranked the 20-th among all 26 runs.

### 7.7. LasigBioTM (Ruas et al., 2020)

The LasigBioTM team participated in all three tasks. Their system for Task 1 was developed by fine-tuning the BioBERT NER model using the training set plus half of the development set, and using the rest of the dev set as validation set for hyper-parameter tuning. For Task 2, they combined the BioBERT NER model and BioBERT Relation Extraction (RE) model, where the NER model was used for trigger word detection and BioBERT RE model was used for classifying relations between trigger words and named entities. The LasigBioTM team submitted three runs, one run for each task. Their run for Task 1 was ranked the 5-th among all 26 runs. Their runs submitted for Tasks 2 and 3 had technical issues and were not included in the final leaderboard, unfortunately.

### 7.8. Melaxtech (Wang et al., 2020)

The Melaxtech team participated in all three tasks. Their systems for all tasks were based on the same core model, named Patent_BERT. The Patent_BERT model is a BioBERT pre-trained language model that was trained further using the patent snippets released by ChEMU on the task of masked language modeling. For Task 1, they fine-tuned Patent_BERT using the Bi-LSTM-CRF (Ma and Hovy, 2016) model. For Task 2, they adopted a two-staged approach: they first followed the approach for Task 1 to detect trigger words. Afterwards, another binary classifier was built by fine-tuning Patent_BioBERT to determine relations between event triggers and named entities in the same sentence. For Task 3, they combined their models in Tasks 1 and 2 to form a pipeline system to produce end-to-end predictions. Melaxtech submitted three runs to each task. They were ranked the 1-st in all three tasks.

### 7.9. Summary of Participants' Approaches

Different approaches were explored by the participating teams. In Table 11, we summarize the key strategies in terms of three aspects: tokenization method, token representations, and core model architecture.

**Table 11:** Summary of participants' approaches.

**Characteristics**	**(Copara et al., 2020b)**	**(Dao and Nguyen, 2020)**	**(Köksal et al., 2020)**	**(Lowe and Mayfield, 2020)**	**(Mahendran et al., 2020)**	**(Pattabhi et al., 2020)**	**(Ruas et al., 2020)**	**(Wang et al., 2020)**
**TOKENIZATION**								
Rule-based	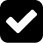	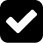	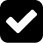	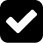	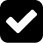	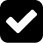	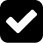	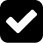
Dictionary-based				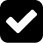				
Subword-based	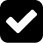		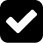				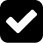	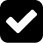
Chemistry domain-specific		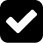		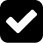				
**REPRESENTATION**								
**Embeddings**								
Character-level		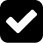			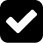			
Pre-trained	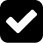	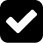	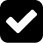		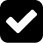		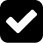	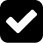
Chemistry domain-specific		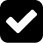	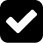		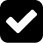		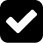	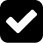
**Features**								
PoS				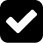		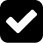		
Phrase				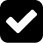		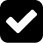		
**MODEL ARCHITECTURE**								
Transformer	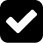		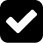				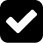	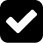
Bi-LSTM		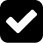			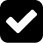			
CNN	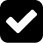	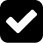			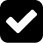			
MLP						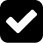		
CRF	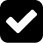	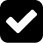			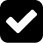	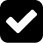		
FSM				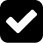				
Rule-based			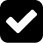	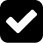	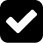			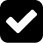

*BiTeM (Copara et al., 2020b); VinAI (Dao and Nguyen, 2020); BOUN-REX (Köksal et al., 2020); NextMove/Minesoft (Lowe and Mayfield, 2020); NLP@VCU (Mahendran et al., 2020); AU-KBC (Pattabhi et al., 2020); LasigBioTM (Ruas et al., 2020); and Melaxtech (Wang et al., 2020)*.

For teams who participated in Tasks 2 and 3, a common two-step strategy was adopted for relation extraction: (1) identify trigger words; and (2) extract the relation between identified trigger words and entities. The first step is essentially an NER task, and the second step is a relation extraction task. As such, NER models were used by all these teams for Tasks 2 and 3 as well as by the teams participating in Task 1. Therefore, in what follows, we first discuss and compare the approaches of all teams without considering the target tasks, subsequently considering relation extraction approaches.

#### 7.9.1. Tokenization

Tokenization is an important data pre-processing step that splits input texts into words/subwords, i.e., tokens. We identify three general types of tokenization methods used by participants: (1) rule-based tokenization; (2) dictionary-based tokenization; and (3) subword-based tokenization. Specifically, rule-based tokenization applies pre-defined rules to split texts into tokens. The rules applied can be as simple as “white-space tokenization,” but can also be a complex mixture of a set of carefully designed rules (e.g., based on language-specific grammar rules and common prefixes). Dictionary-based tokenization requires the construction of a vocabulary and the text splitting is performed by matching the input text with the existing tokens in the constructed vocabulary. Subword-tokenization allows a token to be a sub-string of a word, i.e., subword units, and thus provides a way of handling out-of-vocabulary words. A subword tokenizer learns a set of common subwords based on the word distribution of the training corpus. Thereafter, it can encode rare words by splitting them into meaningful subword units. Popular subword tokenization methods include WordPiece (Schuster and Nakajima, 2012) and Byte Pair Encoding (BPE) (Radford et al., 2019). For each participating team, we consider whether their approach belong to one or multiple of the three categories, and summarize our findings in Table 11. Finally, we also indicate whether their tokenization methods consider domain-specific knowledge in Table 11.

Four teams utilized tokenization methods that are purely rule-based. Specifically, VinAI used the Oscar4 tokenizer (Jessop et al., 2011). This tokenizer is particularly designed for chemical texts, and is made up of a set of pattern matching rules (e.g., prefix matching) that are designed based on domain knowledge from chemical experts. NLP@VCU used spaCy tokenizer,^23^ which consists of a collection of complex normalization and segmentation logic and has been proven to work well with general English corpus. NextMove/Minesoft used a combination of the Oscar4 and LeadMine (Lowe and Sayle, 2015) tokenziers. LeadMine was first run on untokenized text to identify entities using auxiliary grammars or dictionaries. Oscar4 was then used for general tokenization but is adjusted so that each entity recognized by LeadMine corresponds to exactly one token. Four teams, BiTeM, BOUN-REX, LasigBioTM, and Melaxtech, chose to leverage the pre-trained model BERT (or variants of BERT) to address our tasks, and thus, the four teams used the subword-based tokenizer, WordPiece, that is built-in within BERT. BOUN-REX, LasigBioTM, and Melaxtech used BioBERT model which is language model pre-trained on biomedical texts. Since this model is a continual training based on the original BERT model, the vocabulary used in BioBERT does not differ from BERT, i.e., domain-specific tokenization is not used. However, since Melaxtech performed a pre-tokenization using a toolkit CLAMP (Soysal et al., 2018), we consider their approach as domain-specific, since CLAMP is tailored for clinical texts. BiTeM used the model ChemBERTa that is pre-trained on the ZINC corpus. It is unclear yet whether the tokenization is domain-specific due to the lack of documentation of ChemBERTa. Finally, since WordPiece needs an extra pre-tokenization step, we consider it as a hybrid of rule-based and subword-based method.

#### 7.9.2. Representations

When transforming tokens into machine-readable representations, two types of methods are used: (1) feature extraction that represents tokens with their linguistic characteristics, such as word-level features (e.g., morphological features) and grammatical features (e.g., PoS tags); and (2) embedding methods in which token representations are randomly initialized as numerical vectors (or initialized from pre-trained embeddings) and then learned (or fine-tuned) from provided training data. Two teams, NextMove/Minesoft and AU-KBC adopted the first strategy and the other teams adopted the second strategy. Among the teams that used embeddings to represent tokens, two teams, VinAI and NLP@VCU further added character-level embeddings to their systems. All of these six teams used pre-trained embeddings, and five teams used embeddings that are pre-trained for related domains: VinAI and NLP@VCU used the embeddings that are pre-trained on chemical patents (Zhai et al., 2019), BOUN-REX and LasigBioTM used the embeddings from BioBERT that are pre-trained on PubMed corpus. MexlaxTech also used embeddings from BioBERT, but they further tuned the embeddings using the patent documents released in the test phase.

#### 7.9.3. Model Architecture

Various architectures were employed by participating teams. Four teams, BiTeM, BOUN-REX, LasigBioTM, and Melaxtech, developed their systems based on transformers (Vaswani et al., 2017). BiTeM submitted an additional run using an ensemble of a Transformer-based model and a CNN-based model. They also had a third run that is built based on CRF. The other two teams Melaxtech and BOUN-REX added rule-based techniques into their systems. Melaxtech added several pattern-matching rules in their post-processing step. BOUN-REX focused on Task 2 and their system used rule-based methods to determine the event type of each detected event. Two teams, VinAI and NLP@VCU, used the architecture of BiLSTM-CNN-CRF for Task 1. NLP@VCU also participated in Task 2 and they proposed two systems based on rules, and a CNN architecture, respectively. NextMove/Minesoft utilized Chemical Tagger (Hawizy et al., 2011), a model based on Finite State Machine (FSM), and a set of comprehensive rules are applied to generate predictions. AU-KBC proposed two systems for Task 1, based on multi-layer perceptron and CRF, respectively.

#### 7.9.4. Approaches to Relation Extraction

Four of the above teams participated in Task 2 or Task 3. As mentioned before, these teams utilized their NER models for trigger word detection. Thus, here, we only discuss their approaches for relation extraction assuming that the trigger words and entities are known.

NextMove/Minesoft again made use of ChemicalTagger for event extraction. ChemicalTagger is able to recognize WORKUP and REACTION_STEP words, thus, assignment of relationships was achieved by associating all entities in a ChemicalTagger action phrase with the trigger word responsible for the action phrase. A set of post-processing rules were also applied to enhance the accuracy of ChemicalTagger.

LasigBioTM, NLP@VCU, and Melaxtech formulated the task of relation extraction as a binary classification problem. That is, given each candidate pair of trigger word and named entity that co-locate within an input sentence, the goal of the task is to determine whether the candidate pair of entities are related or not.

LasigBioTM developed a BioBERT-based model to accomplish this classification. The input of BioBERT is the sentence containing the candidate pair but the trigger word and named entity of the candidate pair were replaced with the tags “@TRIGGER$” and “@LABEL$,” respectively. The output of BioBERT is modified as a binary classification layer which aims to predict the existence of relation for the candidate pair.

NLP@VCU proposed two systems for relation extraction. Their first system is a rule-based system. Given a named entity, a relation is extracted between the named entity and its nearest trigger word. Their second system is developed based on CNNs. They split the sentence containing the candidate pair into five segments: the sequence of tokens before/between/after the candidate pair, the trigger word, and the named entity of the candidate pair. Separate convolutional units were used to learn the latent representations of the five segments, and a final output layer was used to determine if the candidate pair is related or not.

Melaxtech continued the use of the BioBERT model re-trained on the patent texts released during the test phase. Similar to LasigBioTM, the input to their model is the sentence containing the candidate pair but only the candidate named entity is generalized by its semantic type in the sentences. Furthermore, rules were also applied in the post-processing step to recover long distance relations, including relations across clauses and across sentences.

### 7.10. Summary of Observations

The various approaches adopted by teams and the resulting performances have provided us with valuable experiences in how to address the tasks and what choices of methods are more suitable for our tasks.

#### 7.10.1. Tokenization

In general, domain-specific tokenization tools perform better than tokenization methods that work for general English corpora. This is as expected since the vocabulary of chemical patents contains a large number of domain-specific terminology, and a machine can better understand and learn the characteristics of input texts if the texts are split into meaningful tokens. Another observation is that subword-based tokenization may contribute to overall accuracy. Chemical names are usually long, which make subword-based tokenization a suitable method for breaking down long chemical names. But further investigation is needed to support this claim.

#### 7.10.2. Representation

Pre-trained embeddings are shown to be effective in enhancing system performances. Specifically, the Melaxtech and Lasige_BioTM systems are based on BioBERT (Lee et al., 2020) and ranked the first and third place in Task 1. The VinAI system leveraged embeddings pre-trained on chemical patents (Zhai et al., 2019) and ranked second place. Character-level embeddings are also beneficial, shown by the ablation study in Dao and Nguyen (2020) and Mahendran et al. (2020).

#### 7.10.3. Model Architecture

The most popular choice of model is BERT (Devlin et al., 2019), which is based on Transformer (Vaswani et al., 2017). The model has demonstrated its effectiveness in sequence learning again. The Melaxtech system adopted this architecture and ranked first place in all three tasks. However, it is also worthwhile to note that the architecture of BiLSTM-CNN-CRF is still very competitive w.r.t. BERT. The VinAI system ranked the first place in F_1_-score when relaxed-match is used.

## 8. Error analysis

We perform an error analysis on the test set to understand common errors in different tasks.

### 8.1. Task 1—NER

**Figure 8:**
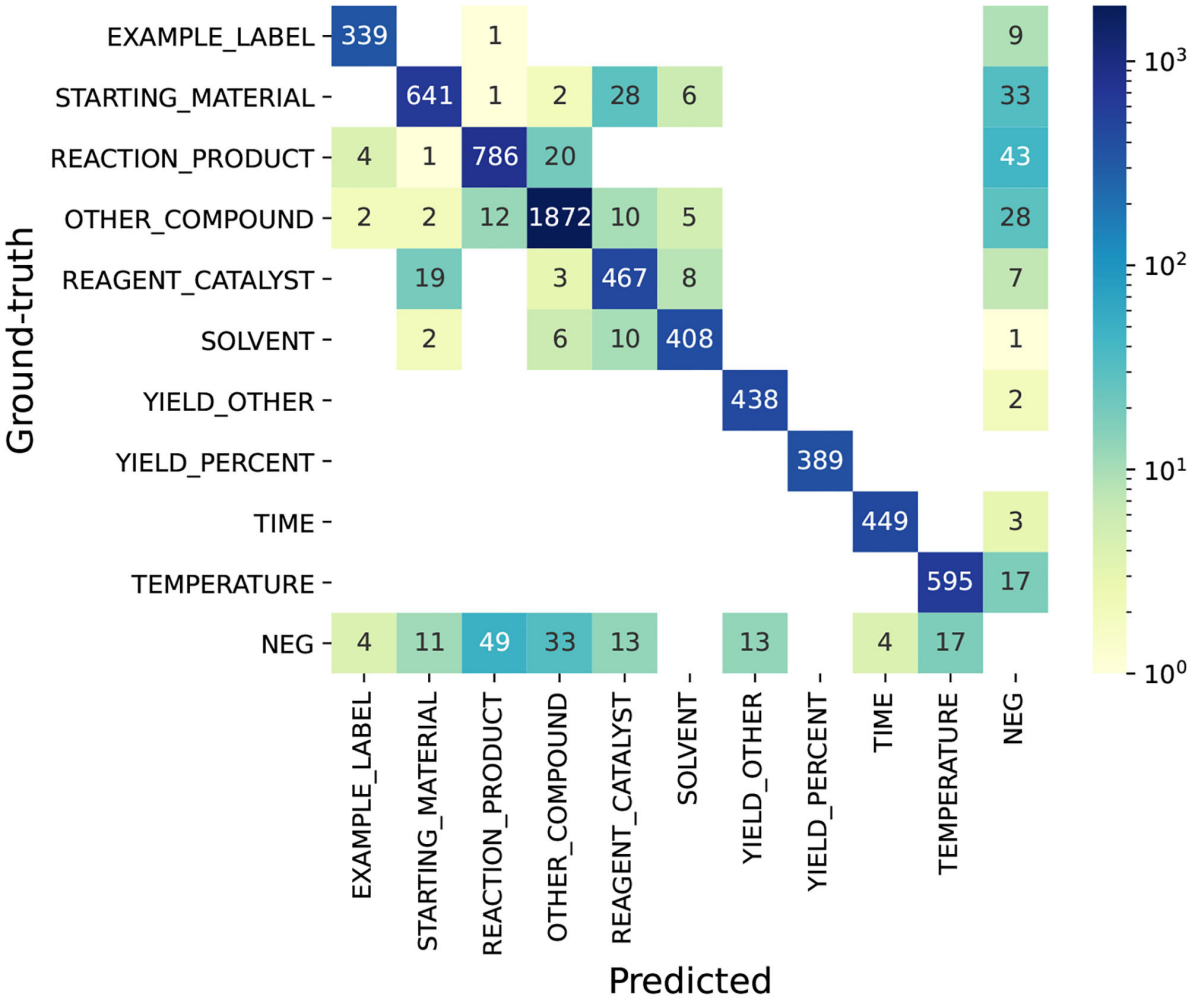
Confusion matrix of the top system on Task 1—NER. NEG (negative entity): ground-truth (or predicted) entities whose text spans are not annotated as entities in the predicted (or ground-truth) set. This confusion matrix is computed under the scenario of exact span-matching.

To understand the errors in Task 1—NER, we use the top-ranking system as an example. We present the confusion matrix of the top system on the test set in Figure 8. The figure shows that the ambiguity between chemical entities is much higher than non-chemical entities. Considering the predicted entities that have labels in the ground-truth set, 141 chemical entities are assigned with wrong labels, while none of non-chemical entities are assigned with wrong labels. In particular, we find it most difficult for the system to tell the differences between STARTING_MATERIAL and REAGENT_CATALYST, REACTION_PRODUCT and OTHER_COMPOUND, and SOLVENT and REAGENT_CATALYST, which correspond to 47 (28 + 19) errors, 32 (20 + 12) errors, and 18 (8 + 10) errors out of the total 141 errors. Next, we present some examples of these errors.

#### 8.1.1. STARTING_MATERIAL vs. REAGENT_CATALYST

**Figure 9:**
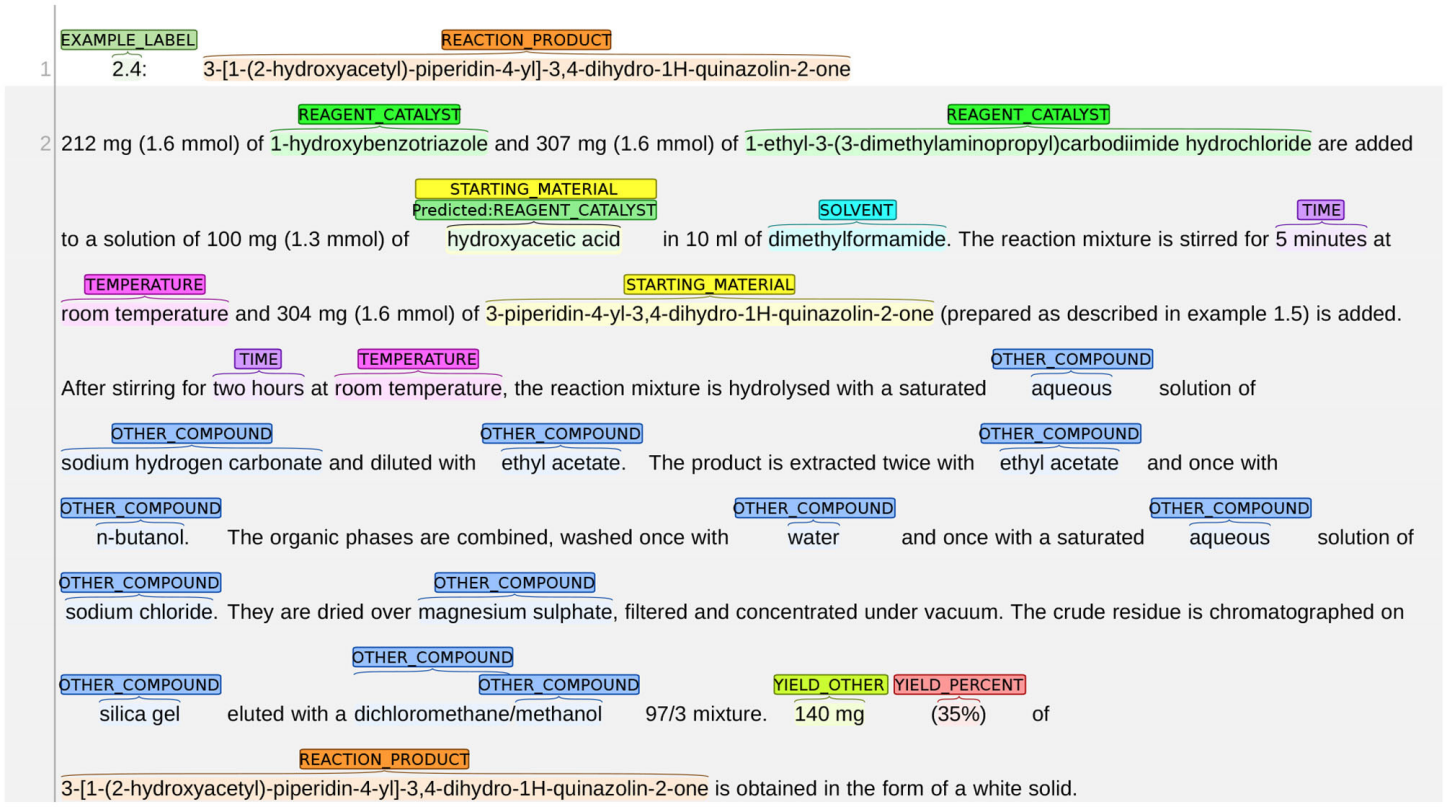
An example of the system misclassifying STARTING_MATERIAL as REAGENT_CATALYST.

An example of the system misclassifying STARTING_MATERIAL as REAGENT_CATALYST is shown in Figure 9. The snippet in this figure describes the synthesis process of the reaction product “3-[1-(2-hydroxyacetyl)-piperidin-4-yl]-3,4-dihydro-1H-quinazolin-2-one.” Given that the chemical expression of the reaction product contains the subword “hydroxyacetyl,” it can be deducted that “hydroxyacetic acid” (line 3) is the starting material since *it provides atoms to the reaction product*. However, the system labels hydroxyacetic acid as REAGENT_CATALYST.

#### 8.1.2. REACTION_PRODUCT vs. OTHER_COMPOUND

**Figure 10:**
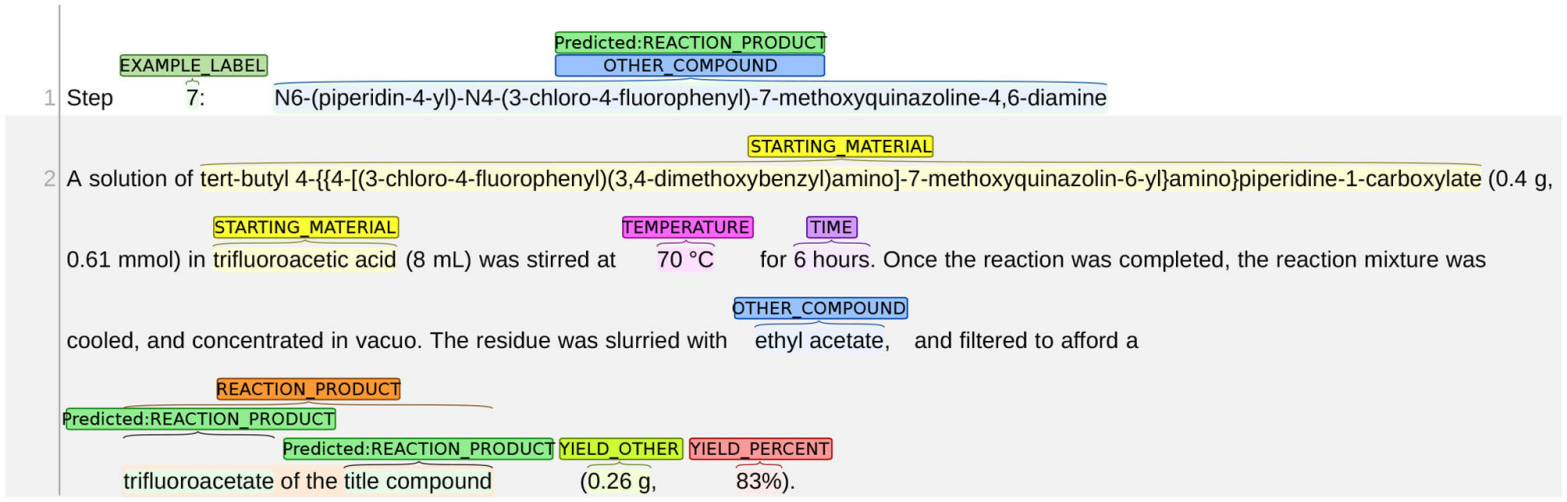
An example of misclassification between REACTION_PRODUCT and OTHER_COMPOUND.

We present an example of the misclassification between REACTION_PRODUCT as OTHER_COMPOUND in Figure 10. The title of this snippet is the name of the chemical compound *N6-(piperidin-4-yl)-N4-(3-chloro-4-fluorophenyl)-7-methoxyquinazoline-4,6-diamine*, but the following chemical reaction describes the synthesis process for preparing *the trifluoroacetate of the title compound*. Thus, in this chemical reaction, the reaction product is no longer the title compound but *the trifluoroacetate of the title compound*. As such, the text span “N6-(piperidin-4-yl)-N4-(3-chloro-4-fluorophenyl)-7-methoxyquinazoline-4,6-diamine” in the first line should be labeled as OTHER_COMPOUND. The system made two mistakes: (1) mislabeled the title compound as REACTION_PRODUCT; and (2) failed to identify the correct span of the reaction product. Both mistakes show that the system is not able to understand the word “trifluoroacetate” correctly. Here, “trifluoroacetate” is not an independent chemical compound but an adjective implying that a different variant of the title compound is obtained. Thus, the word “trifluoroacetate” should not be labeled as an independent reaction product. The word also implies that the actual reaction product is different from the title compound. Thus, the title compound should not be labeled as REACTION_PRODUCT.

#### 8.1.3. SOLVENT vs. REAGENT_CATALYST

**Figure 11:**
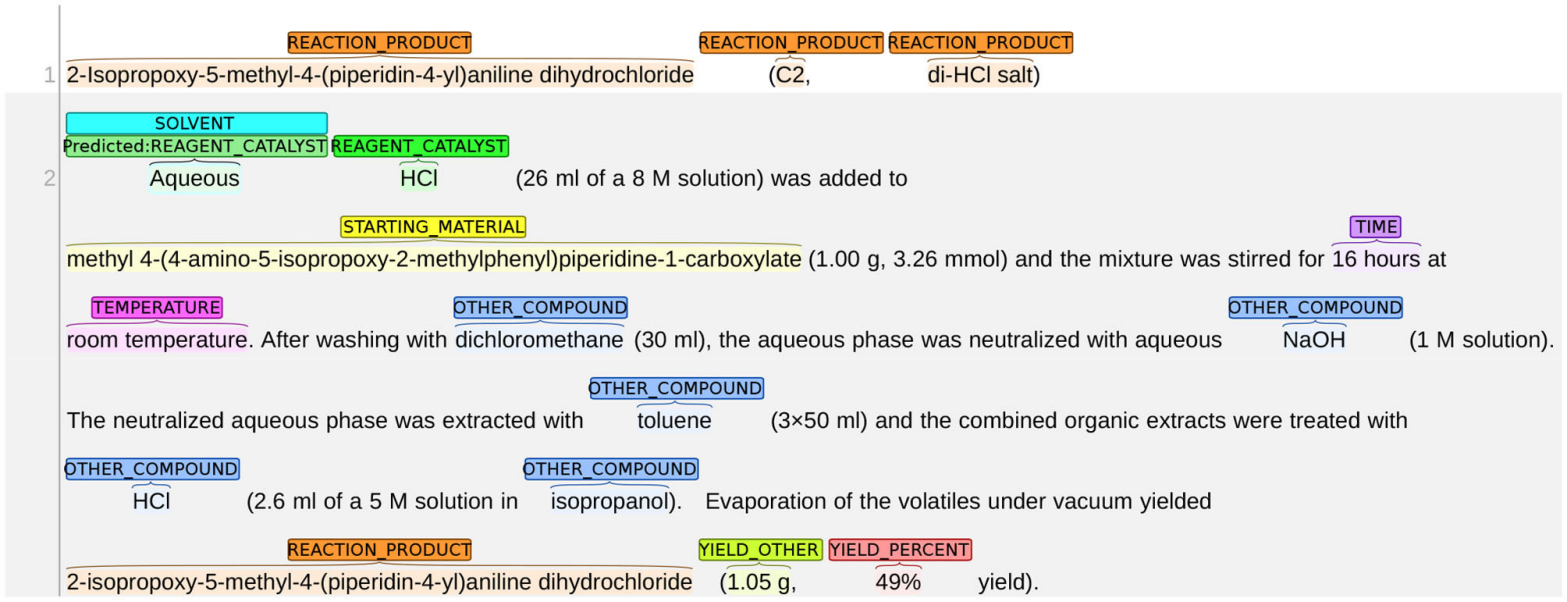
An example of the system misclassifying REAGENT_CATALYST as SOLVENT.

We present an example of the system misclassifying SOLVENT as REAGENT_CATALYST in Figure 11. In most training instances, the role of SOLVENT is implied by expressions, such as “A dissolved in B” or “a solution of A in B.” In this figure, however, the sentence structure changes slightly: the solute (HCI) and solvent (aqueous) are consecutive words with no preposition phrases (e.g., in) between them. The role of SOLVENT is instead implied by the expression within the brackets, i.e., “26 mL of a 8 M solution.” In this case, the system failed to understand the role of “Aqueous” and mislabels it as REAGENT_CATALYST.

### 8.2. Task 2—EE

**Figure 12:**
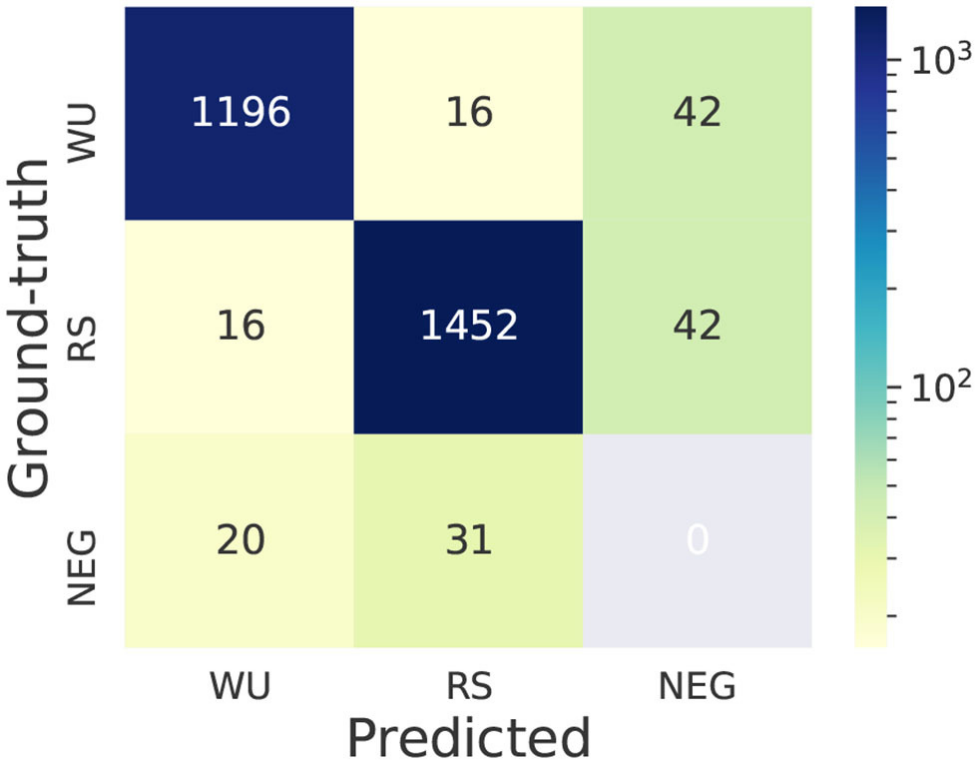
Confusion matrix of the top system for trigger word prediction in Task 2—EE. WU: WORKUP. RS: REACTION_STEP. NEG (negative trigger word): ground-truth (or predicted) trigger words whose text spans are not annotated as trigger words in the predicted (or ground-truth) set. This confusion matrix is computed under the scenario of exact span-matching.

Most teams broke down this task to two sub-tasks: trigger word identification and predicting relations between trigger words and entities. We first investigate the typical errors for trigger word prediction in Task 2. We present the confusion matrix of the top ranking system in Figure 12. The figure shows that more errors (154 errors) are related to trigger word detection and a relatively smaller number of errors (32 errors) are related to trigger word classification. Next we present some examples of different types of errors.

#### 8.2.1. Trigger Word Classification

**Figure 13:**
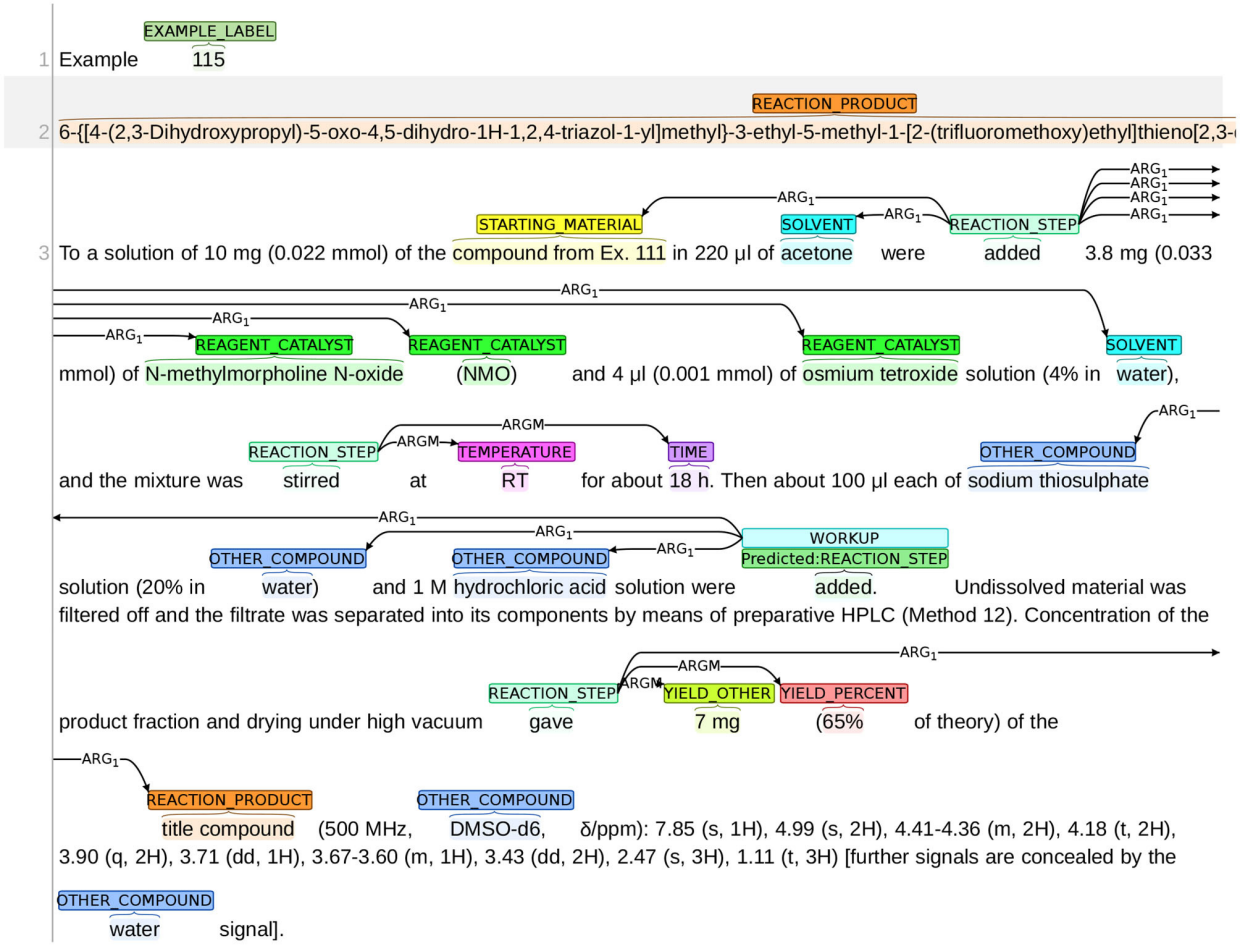
An example of misclassifying WORKUP as REACTION_STEP.

We present an example, where the system misclassified a REACTION_STEP as a WORKUP in Figure 13. In this figure, the word “added” (line 6) is predicted as REACTION_STEP but its true label is WORKUP. If we look at its next sentence, we can see that the action of adding sodium thiosulphate solution and hydrochloric acid solution is to dissolve the material so that the desired components can be filtered out. Thus, the word “added” is part of the procedure to isolate the product, and needs to be labeled as WORKUP. We suspect that this error was caused by sentence segmentation in data preprocessing, since the information needed to make correct decision is only provided by the next sentence. If the two sentences were separated and were not fed into the system simultaneously, the system could not make the correct prediction.

#### 8.2.2. Trigger Word Detection

**Figure 14:**
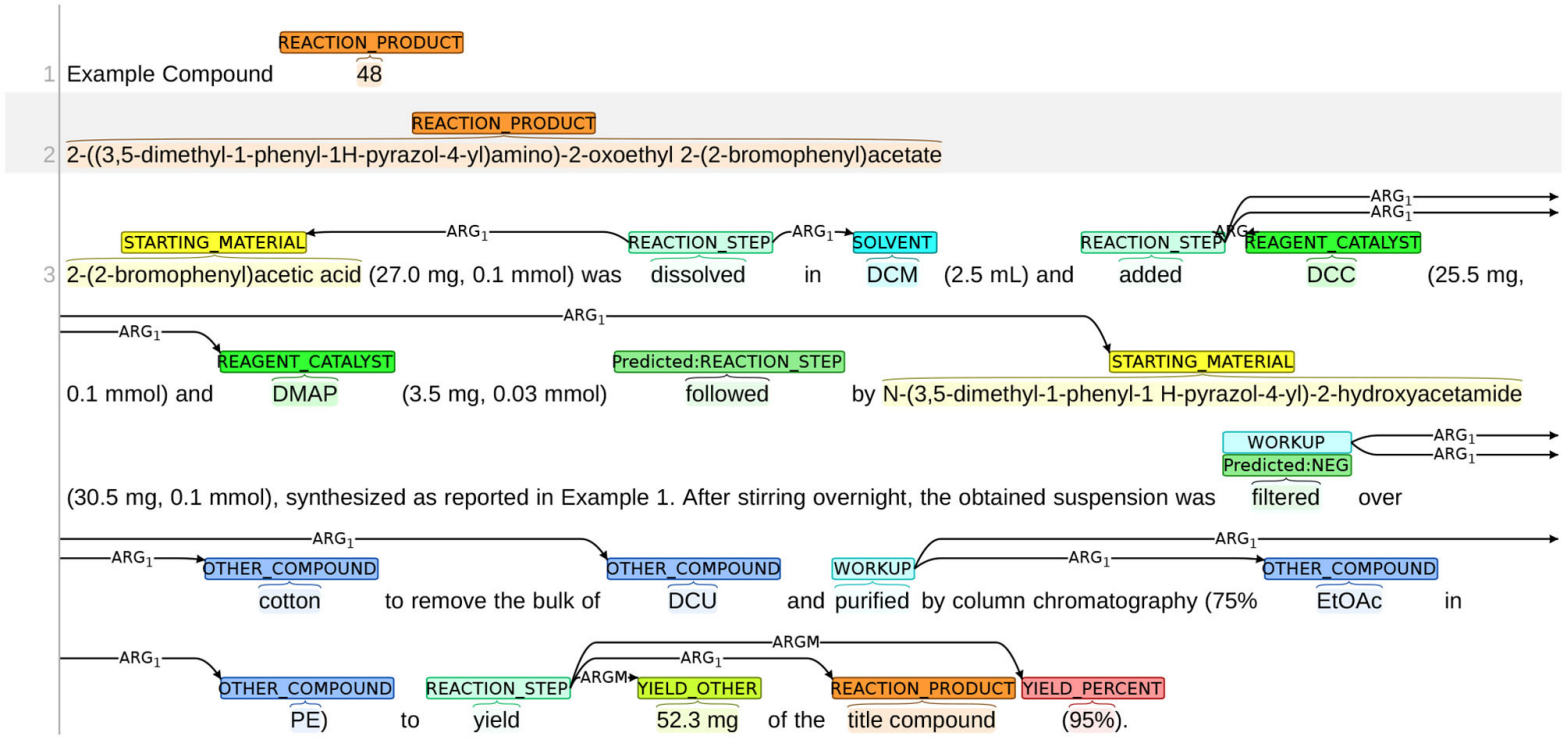
False positive and false negative examples.

We present an example of false positives/negatives in trigger word detection in Figure 14. In this chemical reaction, the starting material 2-(2-bromophenyl)acetic acid was dissolved in DCM, and the solution was added with three chemicals: (1) DCC; (2) DMAP; and (3) N-(3,5-dimethyl-1-phenyl-1 H-pyrazol-4-yl)-2-hydroxyacetamide. Here, the three chemicals should be all associated with the reaction step “added” and the expression “followed by” is only to describe the order of the three chemicals being added. However, the system mislabels the word “followed” as a REACTION_STEP, leading to a false positive error. The work-up procedure for purifying the chemical compounds contains two steps. First, the obtained suspension was filtered. Then the filtrate was purified. The system fails to detect the first step, leading to a false negative error.

#### 8.2.3. Relation Prediction

**Figure 15:**
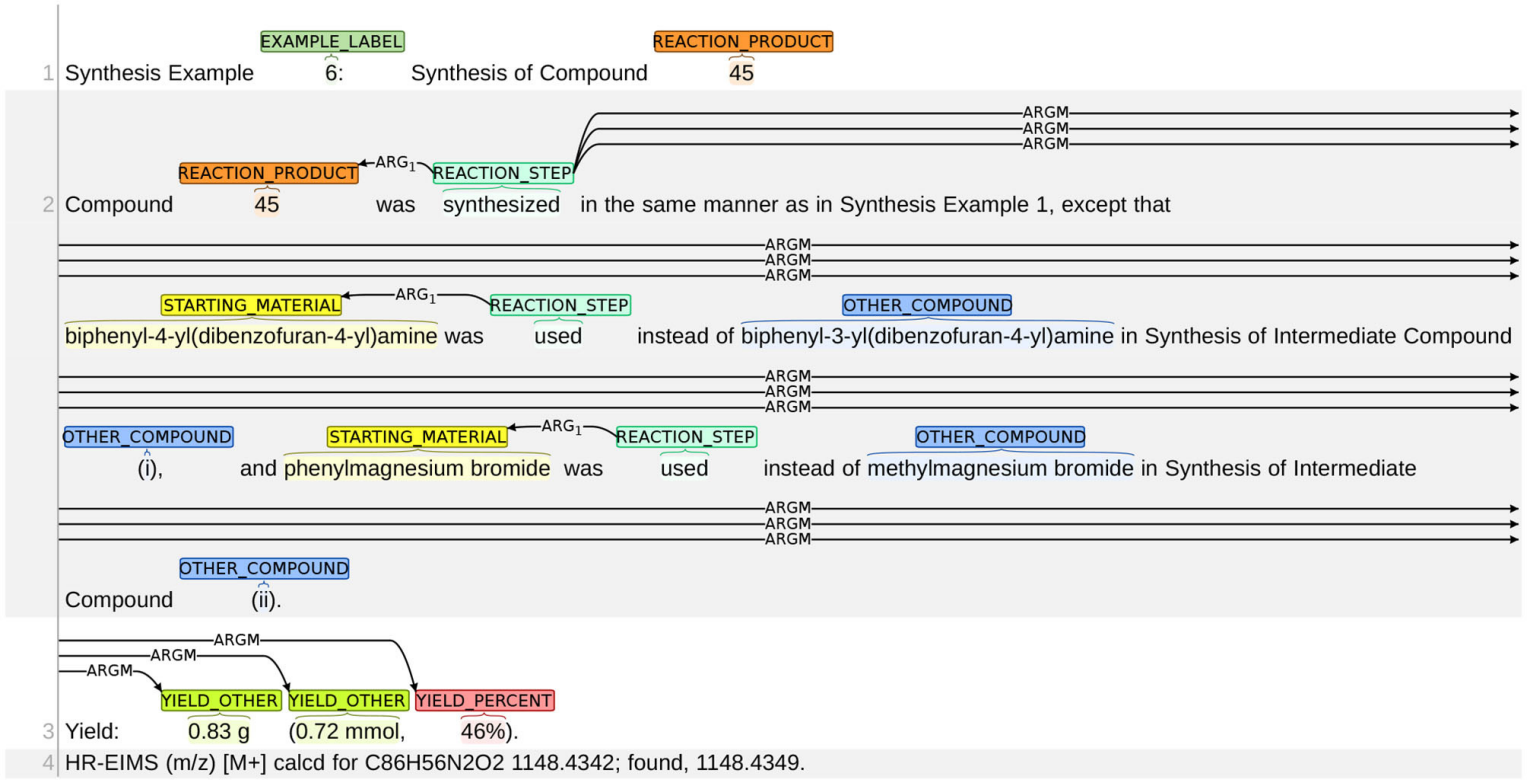
An example of errors in relation prediction. Only the ground-truth labels are included in the figure. In terms of relation prediction, the top ranking system misses three relations: (1) synthesized → 0.83 g; (2) synthesized → 0.72 mmol; (3) synthesized → 46%.

The most significant challenge in relation prediction lies in the fact that many related entities are distant to each other. Figure 15 gives an example. The word “synthesized” (line 2) is related with the chemical “45” (line 2) and the yield entities “0.83 g,” “0.72 mmol,” and “46%” (line 6). However, the system only discovered the relation between “synthesized” and “45” and missed all other three relations, since the three yield entities are very far from the word “synthesized.”

## 9. Discussion of Results

The ChEMU2020 workshop at CLEF was held during 22–26 September 2020. Worldwide participants attended the workshop and presented their systems for the tasks. During the discussion session, the Nextmove Software/Minesoft team contributed an important observation: some pairs of training and test snippets are sampled from the same source document, which results in high similarity in their contents.

In the meantime, the issue of “data leakage” in existing NLP shared tasks and benchmark datasets has been raised in the NLP community. The data leakage here is not limited to direct leakage of training data, where training instances are repeated in the test set, but is extended to include those more general scenarios where testing instances have significant overlap with the training instances, e.g., the overlap due to the same source documents.

Solutions for controlling data leakage are still under exploration. However, the extent of data leakage needs to be taken into consideration when we interpret our evaluation results. That is, when there is significant similarity between train and test data, models that have huge capacity to memorize training instances are more advantageous than others. On the contrary, a test set that has low similarity with the training set will promote those models that go deep in the training data and learn the knowledge required to generalize. Elangovan et al. (2021) present a study on the data leakage issue in various existing benchmark datasets, including the ChEMU 2020 data, and show that unconscious data leakage may lead to inflated evaluation results, i.e., inadvertently interpreting a model's ability to memorize as the ability to generalize.

In this section, we provide an extensive study on the train-test overlaps in our ChEMU chemical reaction corpus, and its impact on our evaluation results in Task 1. We investigate three forms of data leakage: (1) leakage caused by texts being from the same source patents; (2) leakage caused by similar text inputs; and (3) leakage caused by similar target entities.

### 9.1. Impact of Source Patents

**Figure 16:**
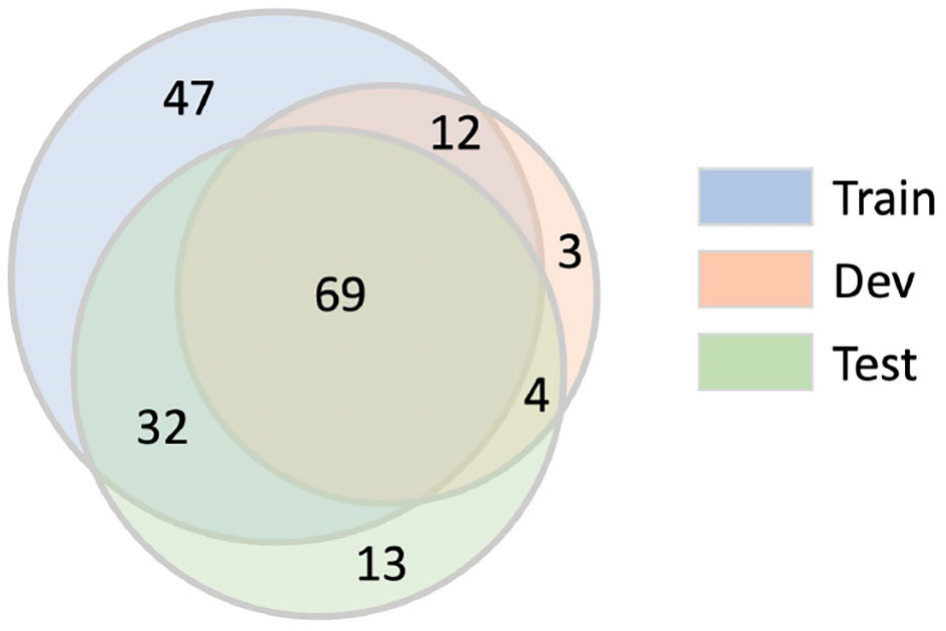
Number of unique source patents in training, development, and test set. Each number represents the number of unique source patents within the sector partitioned by the gray circle boundaries.

Patent snippets extracted from the same source patent may be more similar in terms of linguistic properties, such as their vocabulary, sentence structures, and topical distributions. The snippets in our corpus are sampled from a relatively small set of source patents: 1,500 snippets sampled from 180 patents. The unique source patents that are used in the training, development and test sets are summarized in Figure 16. There are 180 unique source patents used in the training set, out of which 101 overlap with the test set, 81 overlap with the development set, and 69 overlap with both the test and development sets. There are 13 source patents in the test set that have not been used in either the train or dev snippets. These 13 source patents have been used to generate 14 patent snippets in the test set.

Let $T_{new}$ represents the set of 14 patent snippets with new source patents. We evaluate all runs on Task 1 using $T_{new}$ and report their F_1_-scores in the column “F” of Table 12. We also evaluate the runs by only considering the compound entities in $T_{new}$ and report their F_1_-scores in the column “F^*^.” We also present their performance change on $T_{new}$ compared with those on the original test set by measuring their (columns “ΔF,” “ΔF^*^,” “%,” and “%^*^”).

**Table 12:** Evaluation results on $T_{new}$.

**Run**	**F**	**ΔF**	**%**	F^*^	ΔF^*^	%^*^
Melaxtech-run1	0.8777	−0.0793	8.3	0.8672	−0.0766	8.1
Melaxtech-run2	0.8721	−0.0837	8.8	0.8521	−0.0903	9.6
Melaxtech-run3	0.8777	−0.0764	8.0	0.8672	−0.0732	7.8
VinAI-run2	0.8696	−0.0825	8.7	0.8477	−0.0932	9.9
VinAI-run1	0.8644	−0.0789	8.4	0.8448	−0.0903	9.7
LasigBioTM-run1	0.8525	−0.0867	9.2	0.8333	−0.0859	9.3
BiTeM-run3	0.8517	−0.0713	7.7	0.8238	−0.0689	7.7
BiTeM-run2	0.8345	−0.0753	8.3	0.8163	−0.0587	6.7
NextMove/Minesoft-run1	0.7652	−0.1331	14.8	0.7277	−0.1373	15.9
NextMove/Minesoft-run2	0.7652	−0.1325	14.8	0.7277	−0.1365	15.8
NLP@VCU-run1	0.7729	−0.0929	10.7	0.7744	−0.0940	10.8
KFU_NLP-run1	0.8242	−0.0407	4.7	0.8338	−0.0398	4.6
NLP@VCU-run2	0.7698	−0.0904	10.5	0.7629	−0.0982	11.4
NLP@VCU-run3	0.7941	−0.0648	7.5	0.8052	−0.0539	6.3
KFU_NLP-run2	0.8036	−0.0416	4.9	0.8040	−0.0397	4.7
NextMove/Minesoft-run3	0.6717	−0.1464	17.9	0.6296	−0.1451	18.7
KFU_NLP-run3	0.7957	−0.0154	1.9	0.8041	0.0042	0.5
BiTeM-run1	0.6984	−0.1072	13.3	0.6275	−0.1074	14.6
OntoChem-run1	0.5446	−0.1373	20.1	0.4615	−0.1520	24.8
AUKBC-run1	0.5188	0.0103	2.0	0.5439	−0.0815	13.0
AUKBC-run2	0.3654	0.0903	32.8	0.3846	0.0253	7.0
SSN_NLP-run1	0.2203	−0.0108	4.7	0.2746	−0.0080	2.8
SSN_NLP-run2	0.2159	−0.0148	6.4	0.2736	−0.0090	3.2
JU_INDIA-run1	0.2393	0.1352	>100	0.2963	0.1580	>100
JU_INDIA-run2	0.0450	0.0244	>100	0.0562	0.0265	89.2
JU_INDIA-run3	0.0450	0.0244	>100	0.0562	0.0265	89.2

F: F_1_-scores; ΔF: change in F_1_-scores (compared with Table 6); %: percentage of absolute change in F_1_-scores; F^*^: F_1_-scores on COMPOUND entities; ΔF^*^: change in F_1_-scores on COMPOUND entities (compared with Table 6); %^*^: percentage of absolute change on COMPOUND entities.

As shown by the column“ΔF”, most runs are found to have drops in performance. But the general trend is that the runs with higher rankings are more robust on the new test set compared with those with lower rankings. The top five runs show a fairly consistent drop in F_1_-score of ~0.08 (a ratio of ~8.0%). The lower ranking runs seem to have much higher variances, and the maximum change observed reaches more than 100%. There are some runs that do not follow this trend, for example, the runs submitted by KFU_NLP, SSN_NLP, and NextMove/Minesoft. The runs submitted by KFU_NLP show very small performance changes (<5%), and so do SSN_NLP runs. The runs submitted by NextMove/Minesoft were found to have more performance changes on $T_{new}$, considering their ranking in Table 6. But more investigations are needed to confirm the reasons behind these observations.

### 9.2. Impact of Input Text Similarity

The vocabulary similarity between train and test instances in terms of the input text also impact model performances. Models with high memorization capacity may gain more benefits when the input texts of test instances are significantly similar with those of training instances. Herein, we investigate the impact of text similarity by dividing the original test set into several subsets, each of which has a different level of similarity with the training set, and observe the performances of all runs on these subsets.

#### 9.2.1. Text Similarity Computation

For each test snippet, we choose the training snippet that is most similar with the test snippet, and use similarity between the two snippets to represent the similarity of the test snippet with the entire training set. To quantify the similarity between two snippets, we convert their input texts into their bag-of-words vector representations. Then the similarity is computed as the cosine similarity between the two bag-of-words. As a result, the similarity value between two snippets is within the range [0, 1] and higher value indicates higher similarity between two snippets.

We split the test snippets into four groups Q1 to Q4, where the snippets within each group have a similarity of [0.0, 0.25), [0.25, 0.5), [0.5, 0.75), and [0.75, 1.0], respectively. Thus, Q1 represents the set of test snippets that are most different from the training set, and Q4 represents the set of test snippets that are most similar to the training set. We find that 10% of test snippets belong to Q2, 60% belong to Q3 and 30% belong to Q4. There are no test snippets with <0.25 similarity with the training set, and thus, Q1 is empty.

The performances on each resultant group are summarized in Table 13. For ease of comparison, for each run, we present its absolute change in F_1_-score on each group of test snippets compared with the original test set, and the percentage of such change. We also present their maximum changes in F_1_-scores across different groups.

**Table 13:** F_1_ scores on Q2-4.

**Run**	**Q2**	**Q2 (%)**	**Q3**	**Q3 (%)**	**Q4**	**Q4 (%)**	**Δ**
Melaxtech-run1	−0.0481	5.0	0.0008	0.1	0.0097	1.0	0.0578
Melaxtech-run2	−0.0462	4.8	−0.0013	0.1	0.0126	1.3	0.0588
Melaxtech-run3	−0.0482	5.1	0.0008	0.1	0.0094	1.0	0.0576
VinAI-run2	−0.0409	4.3	−0.0025	0.3	0.0132	1.4	0.0541
VinAI-run1	−0.0496	5.3	−0.0037	0.4	0.0187	2.0	0.0683
LasigBioTM-run1	−0.0451	4.8	0.0001	0.0	0.0106	1.1	0.0557
BiTeM-run3	−0.0457	5.0	−0.0043	0.5	0.0174	1.9	0.0631
BiTeM-run2	−0.0476	5.2	−0.0059	0.6	0.0209	2.3	0.0685
NextMove/Minesoft-run1	−0.0668	7.4	0.0110	1.2	−0.0042	0.5	0.0778
NextMove/Minesoft-run2	−0.0645	7.2	0.0105	1.2	−0.0036	0.4	0.0750
NLP@VCU-run1	−0.0594	6.9	−0.0086	1.0	0.0279	3.2	0.0873
KFU_NLP-run1	−0.0281	3.2	0.0239	2.8	−0.0367	4.2	0.0606
NLP@VCU-run2	−0.0638	7.4	−0.0098	1.1	0.0312	3.6	0.0950
NLP@VCU-run3	−0.0489	5.7	−0.0110	1.3	0.0300	3.5	0.0789
KFU_NLP-run2	−0.0188	2.2	0.0181	2.1	−0.0280	3.3	0.0461
NextMove/Minesoft-run3	−0.0535	6.5	0.0154	1.9	−0.0147	1.8	0.0689
KFU_NLP-run3	−0.0088	1.1	0.0170	2.1	−0.0280	3.5	0.0450
BiTeM-run1	−0.0596	7.4	−0.0071	0.9	0.0248	3.1	0.0844
OntoChem-run1	−0.0632	9.3	0.0206	3.0	0.0288	4.2	0.0920
AUKBC-run1	−0.0067	1.3	0.0495	9.7	0.0782	15.4	0.0849
AUKBC-run2	0.0559	20.3	0.0080	2.9	0.0885	32.2	0.0805
SSN_NLP-run1	−0.0263	11.4	−0.0015	0.6	0.0090	3.9	0.0353
SSN_NLP-run2	−0.0255	11.1	−0.0036	1.6	0.0122	5.3	0.0377
JU_INDIA-run1	0.1432	137.6	−0.0031	3.0	−0.0238	22.9	0.1670
JU_INDIA-run2	0.0047	22.8	−0.0009	4.4	0.0058	28.2	0.0067
JU_INDIA-run3	0.0047	22.8	−0.0009	4.4	0.0058	28.2	0.0067

*Q1 is omitted since it's empty. Qi: change in F_1_-scores on set Qi; Qi(%): percentage of absolute change on set Qi, where i∈[2, 4]. Δ: maximum change in F_1_-scores across Q2-4*.

The average change in F_1_-scores across Q2-4 (i.e., average value of column “Δ”) is 0.0659. Only the teams NLP@VCU, NextMove/Minesoft, OntoChem, and AUKBC have slightly greater fluctuations across different quartiles. On average, NLP@VCU has a change of 0.0871, NextMove/Minesoft has a change of 0.0739, OntoChem has a change of 0.0920, and AUKBC has a change of 0.0827. But in general, the fluctuations of all runs across different subsets are similar and relatively small.

The results in Table 13 show that different runs have different trends in F_1_-scores across Q2-4. In general, we expect that a machine learning method will benefit from the train-test similarity and there may be consistent increase in F_1_-scores when the test set is switched from Q2 to Q3, and then Q3 to Q4. Indeed, this phenomenon is observed on most runs. However, there are also a few teams that do not follow this distribution. The teams NextMove/Minesoft, KFU_NLP, and OntoChem achieve their best accuracy on Q3.

### 9.3. Impact of Entity Similarity

The overlap between target entities in the test set may also have influence on model performances. In an extreme case where the target entities in the test set are identical with the entities in the training set, a model can perform well by simply memorizing the entities in the training set and performing dictionary look-up when predicting. Therefore, a test set where the entities highly overlap with the training set will promote the models that are powerful in memorization, and misinterpret their capabilities of memorization as their capabilities of capturing deep contextual information in the input.

To quantify the train-test overlap in terms of target entities, a straightforward method is to find out the entities that appear in both training and test set and treat these overlapped entities as “easy entities” that are predictable simply by memorization. However, if an overlapped entity occurs multiple times in the training set and is assigned with different labels in these multiple occurrences, the entity may not be easy to predict by memorization. For example, if an entity “water” occurs 10 times in the training set, and five of them are tagged as “SOLVENT” and the others as “O” (none-entity token in the BIO scheme), simply memorizing the entity “water” is not enough to predict its label. Thus, we quantify the *entity predictability w.r.t. memorization* by computing the information-theoretic entropy of an entity in the training set.

#### 9.3.1. Computation of Entity Entropy

For each entity in the training set, we compute a probability distribution of it being tagged with the 10 entity labels (Table 2). Given an entity *e* with a probability distribution *p*(*e*) = [*p*_1_, …, *l*_*l*_] where *p*_*i*_ represents the probability of *e* being tagged as the *i*th label, the entropy *E*(*e*) of *e* is computed as follows, where *l* represents the total number of labels:

(1) $$\begin{array}{r} E\left(e\right)=-\overset{i=l}{\underset{i=1}{\sum }}p_{i}\log_{10}p_{i} \end{array}$$

The entropy value range of an entity is [0.0, 1.0]. The entropy value of 0.0 refers to the case where the entity is always tagged with the same label, and the entropy value of 1.0 refers to the case where the label of an entity is extremely random. If an entity in the test set does not occur in the training set, we set its entropy as 1.0 since we cannot obtain any information about the entity by memorizing its occurrence(s) in the training set.

We compute the entropy of entities in both the training, development, and test sets. Note that when computing the entropy of all entities, we only consider their occurrences in the training and development set, since we aim to understand the predictability of these entities by memorizing their occurrences in the training and development sets.

There are 2,393 unique entities in the test set, and 1,160 of them do not appear in the training or test set. Among the rest of the entities, 238 of them have an entropy value ranging within [0.25, 1.0), 310 of them have an entropy value ranging within (0.0, 0.25), and 685 of them have the entropy value equal to 0.

Among the entities with an entropy of 0, the entity “title compound” appears as most frequent in the training and development set: all its 388 occurrences are labeled as REACTION_COMPOUND. Another example is the entity “brine,” which occurs 216 times and is always labeled as OTHER_COMPOUND. The entity “methanol” appear a lot in the training and development set, but is much harder to predict (entropy of 0.542), since the diversity of its labels is quite high: 125 of its occurrences are labeled as OTHER_COMPOUND, 66 are labeled as SOLVENT, 37 are labeled as “O” (none-entity), 11 as STARTING_MATERIAL, 10 as REAGENT_CATALYST. Another example is the entity “aqueous” with entropy of 0.412: 261 of its occurrences are labeled as OTHER_COMPOUND, 123 as “O,” 42 as “SOLVENT” and 6 as REAGENT_CATALYST.

We split the entities in the test set into four sets S1 to S4. Set S1 contains the entities with an entropy value of 0.0, S2 contains the entities whose entropy value is within (0.0, 0.25), S3 contains the entities whose entropy value is within [0.25, 1.0), and S4 contains the entities whose entropy value is equal to 1.0. We evaluate all runs on these the four sets. Suppose we are evaluating a model using S1. In this case, we only use the entities in S1 when counting true positives and false negatives. When counting false positives, if an entity predicted by a submitted run has not appeared in the test set, we compute its entropy on the fly. If its entropy lies within the entropy interval of S1, we include it as a false positive, and ignore it otherwise.

The evaluation results of all runs are summarized in Table 14. For ease of comparison, we only show the differences between the F_1_-scores observed in this experiment and the scores reported in **Table 6**. As shown in the table, the F_1_-scores of most runs decrease as the test set changes from S1 to S4. There are not many exceptions, and only AUKBC-run1 performs slightly better on S3 than S2. This is as expected, since entities in S1 have lower information entropy and are easier to predict compared those in other sets.

**Table 14:** F_1_ scores of all runs on S1 to S4.

**Run**	**S1**	**S2**	**S3**	**S4**	**Δ**
Melaxtech-run1	0.0309	0.0084	−0.0047	−0.0329	0.0638
Melaxtech-run2	0.0286	0.0125	0.0003	−0.0424	0.0710
Melaxtech-run3	0.0287	0.0098	−0.0024	−0.0362	0.0649
VinAI-run2	0.0335	0.0101	−0.0066	−0.0359	0.0694
VinAI-run1	0.0384	0.0148	0.0006	−0.0536	0.0920
LasigBioTM-run1	0.0430	0.0251	0.0190	−0.0884	0.1314
BiTeM-run3	0.0345	0.0317	0.0043	−0.0764	0.1109
BiTeM-run2	0.0463	0.0371	0.0153	−0.1006	0.1469
NextMove/Minesoft-run1	0.0595	0.0233	−0.0283	−0.0521	0.1116
NextMove/Minesoft-run2	0.0601	0.0232	−0.0283	−0.0521	0.1122
NLP@VCU-run1	0.0792	0.0469	0.0043	−0.1186	0.1978
KFU_NLP-run1	0.0657	0.0358	−0.0308	−0.0640	0.1297
NLP@VCU-run2	0.0829	0.0495	0.0066	−0.1271	0.2100
NLP@VCU-run3	0.0861	0.0459	0.0026	−0.1203	0.2064
KFU_NLP-run2	0.0922	0.0571	−0.0035	−0.1269	0.2191
NextMove/Minesoft-run3	0.0541	0.0172	−0.0140	−0.0524	0.1065
KFU_NLP-run3	0.0832	0.0849	0.0349	−0.1727	0.2576
BiTeM-run1	0.0604	0.0949	0.0449	−0.2162	0.3111
OntoChem-run1	0.0589	0.0317	0.0050	−0.1062	0.1651
AUKBC-run1	0.0912	0.0426	0.0504	−0.1707	0.2619
AUKBC-run2	0.0611	0.0487	−0.0217	−0.0742	0.1353
SSN_NLP-run1	0.0382	0.0266	0.0439	−0.0500	0.0939
SSN_NLP-run2	0.0535	0.0211	0.0456	−0.0526	0.1061
JU_INDIA-run1	0.0330	0.0501	−0.0138	−0.0659	0.1160
JU_INDIA-run2	0.0071	0.0136	−0.0042	−0.0176	0.0312
JU_INDIA-run3	0.0071	0.0136	−0.0042	−0.0176	0.0312

*S*i*: change in F_*1*_-scores on set S*i*, where *i*∈*[1, 4]*. Δ: maximum change in F_*1*_-scores across four sets*.

The average change in F_1_-scores across S1–4 (i.e., average value of column “Δ”) is 0.1367, which is much higher than the average change of 0.0659 in Table 13. Many more teams have higher fluctuations across S1 to S4 compared to the fluctuations across Q2 to Q4. Moreover, the range of the fluctuations of all teams is [0.0312, 0.2619], which is a much wider change compared with Table 13. This may indicate that models are more sensitive to what they need to predict, compared with what they can use for prediction. The top five runs are relatively more robust against the changes in test sets, given their small fluctuations in both Tables 13, 14.

### 9.4. Summary

In the above experiments, we generate stratified test sets with controlled similarity over the training set. We use these test sets to re-evaluate the runs we received in Task 1 NER and investigate their capability of generalizing on new data. The above experimental results show that the performances of different systems do change significantly with the test sets. Most models perform better on test sets that are more similar with the training set. On the test set in which only the test instances from new source documents are included, the top ten runs in Task 1 have ~10-point drop on average in F_1_-scores compared with the original test set. On the stratified test subsets where the input text similarity is controlled within each subset, the top ten runs have ~6-point difference in F_1_-scores across all subsets. Similar phenomena is also observed on the stratified subsets where the similarity of target entities is controlled.

Although the absolute performances of different runs change with the test set, the ranking of these runs does not change much. For example, the top three teams in Task 1 remain the same across Tables 12–14, with almost the same ranking. This shows that the current test set still correctly reflect the ranking of how each model generalize on new test data.

However, the strikingly different performances of the same model on different test sets show that avoiding unconscious data leakage is still important. We believe that controlling the source documents of the test instances is crucial in our future shared tasks, since a key feature of a good model is its ability to process *unseen* documents. How to avoid other types of data leakage, e.g., train-test overlap in terms of input texts and target entities, and to what extent we need to control such train-test overlaps, remain a question to us. On one hand, having control over the train-test overlaps is crucial when we interpret our evaluation results: we want to know if a model can generalize well on new data. But extreme elimination of train-test overlap may be infeasible or unnecessary. Ultimately, machine learning models are trying to learn representative distributions underlying the data by capturing such similarities and correlations amongst the training data. We are still exploring methods for mitigating/controlling train-test overlaps. But we believe at least using stratified test sets instead of a single test set will provide more comprehensive evaluation results.

## 10. Conclusions

This paper presents an overview of the activities in the ChEMU 2020 evaluation lab. We introduced our motivation of hosting the lab, the tasks provided by the lab, and the evaluation framework used. We also summarized the evaluation results, discussed participants' approaches, and presented analysis of the results.

The ChEMU 2020 evaluation lab was hosted to provide tasks that focus on information extraction over chemical patents. Two key information extraction tasks were provided: named entity recognition, which aims to identify chemical compounds and their specific roles in chemical reactions, and event extraction, which aims to identify the single event steps that form a chemical reaction. A new high-quality chemical reaction corpus annotated by chemical experts was made available to the public. The corpus is annotated with fine-grained chemical entities and the relations between reaction steps and these entities. Analysis of the inter-annotator agreement demonstrates high reliability of the annotation.

The task was held between April 2020 to June 2020. We received registrations from 39 teams, 46 runs from 11 teams, and 8 paper submissions from 8 teams detailing their approaches to address the various tasks. Many effective solutions were reported: the best systems achieved up to nearly 0.98 macro-averaged F_1_-score on the NER task (and up to 0.99 F_1_-score on a relaxed match), 0.95 F_1_-score on the isolated relation extraction task, and around 0.92 F_1_-score for the end-to-end systems. These results strongly outperformed baselines.

Comparison of participants' approaches to the tasks confirmed the effectiveness of pre-trained models/embeddings, and indicate that incorporation of domain-specific knowledge is crucial to model performance. We found that the use of domain-specific tokenizers, such as Oscar4 is beneficial to model performance. We also found that systems that used domain-specific embeddings, such as embeddings trained on biochemical texts performed better than those which used embeddings trained on a general English corpus.

Finally, we investigated how the similarity between the training and test sets affect our evaluation results. We investigated three types of train-test similarities, including similarity in source patents, similarity in the patent texts, and similarity in the target entities. We found that train-test similarity had observable influence on model performances. Most runs that we received show a degradation in model performances when the test set has lower similarity with the training set. However, we observed that grammar-based models may behave differently compared with machine learning models, which are purely data-driven, with our results suggesting that manual tuning of rule-based methods may result in some overfitting. We also confirmed that the top ranking runs were the most robust against the changes in the test set.

The ChEMU 2020 shared task makes an important contribution to progressing the state of the art in automatic extraction of reaction information in chemical patents, with very strong performance exhibited on the key chemical entity recognition and the relations connecting these entities. However, certain limitations in the definition of the current task preclude direct application to the broader full-text chemical patent literature. One such limitation is the reliance on the pre-identified reaction snippets, artificially eliminating significant quantities of additional text in patents from analysis by the model. This pre-segmentation of the full patents clearly simplifies the task by identifying reaction descriptions, thereby eliminating segments potentially confusing to the models. This may explain the high performance of models in the shared task. Another limitation is the restriction to consideration of explicitly mentioned entities, when indirect or generic references abound in these texts.

Both of these limitations will be addressed in the ChEMU 2021 shared task (He et al., 2021). The creation of a gold standard data set of full patents annotated with reaction spans and references between them, building on the work of Yoshikawa et al. (2019), is underway to facilitate subsequent analysis of reaction spans with the models developed for ChEMU 2020. The ChEMU-Ref dataset (Fang et al., 2021b) is also in development to support analysis of anaphoric relations that occur in the reaction texts (Fang et al., 2021a). We are looking forward to increased capabilities for text mining of chemical patents for critical chemical reaction information.

## Data Availability Statement

The Data and Annotation Guidelines for the ChEMU 2020 shared task are available under the CC BY NC 3.0 license on Mendeley Data (Verspoor et al., 2020). The gold standard annotations for the test data will remain “blind” until they are released in late 2021. The evaluation system for the test data will remain open at: http://chemu2020.eng.unimelb.edu.au/ until such time as they are released publicly.

## Author Contributions

JH: managing day-to-day activities of ChEMU lab, evaluation of shared task results, baseline design, and paper writing. DN, ZZ, BF, and HY: data preparation, paper revision, and baseline design. SA, CD, CT, RH, and ZA: data preparation, paper revision, and organization of ChEMU lab. AA: development of submission website. LC, TC, and TB: Contributions to ChEMU team member (staff/student) supervision; paper revision. KV: organization of ChEMU lab, data preparation, baseline design, and paper writing. All authors contributed to the article and approved the submitted version.

## Conflict of Interest

SA, CD, CT, RH, and ZA are employed by the company Elsevier. DN is employed by the company VinAI. HY is employed by the company Fujitsu. The remaining authors declare that the research was conducted in the absence of any commercial or financial relationships that could be construed as a potential conflict of interest.

## Footnotes

^1^https://www.policymed.com/2014/12/a-tough-road-cost-to-develop-one-new-drug-is-26-billion-approval-rate-for-drugs-entering-clinical-de.html.

^2^https://n2c2.dbmi.hms.harvard.edu/.

^3^https://trec.nist.gov/.

^4^https://biocreative.bioinformatics.udel.edu/.

^5^https://2019.bionlp-ost.org/.

^6^https://sites.google.com/site/clefehealth/.

^7^https://chemu.eng.unimelb.edu.au/.

^8^https://pubmed.ncbi.nlm.nih.gov/.

^9^https://go.drugbank.com/.

^10^https://github.com/aspuru-guzik-group/chemical_vae/tree/master/models/zinc.

^11^https://www.nltk.org/api/nltk.tokenize.html.

^12^https://www.reaxys.com Reaxys® Copyright ©2020 Elsevier Limited except certain content provided by third parties. Reaxys is a trademark of Elsevier Limited.

^13^http://brat.nlplab.org/.

^14^https://chemu.eng.unimelb.edu.au/brat/index.xhtml#/chemu_sample/.

^15^https://www.wipo.int/classifications/ipc/en/.

^16^http://chemu.eng.unimelb.edu.au/.

^17^https://bitbucket.org/nicta_biomed/brateval/src/master/.

^18^http://mallet.cs.umass.edu/.

^19^The run that we received from team Lasige_BioTM is not included in the table due to a technical issue found in this run.

^20^The run that we received from the Lasige_BioTM team is not included in the table as there was a technical issue in this run. Two runs from Melaxtech, Melaxtech-run2 and Melaxtech-run3, had very low performance, due to an error in their data pre-processing step.

^21^https://github.com/seyonechithrananda/bert-loves-chemistry.

^22^https://github.com/zenanz/ChemPatentEmbeddings.

^23^https://spacy.io/api/tokenizer.
